# A Comprehensive Review of Somatic and Germline Biomarkers Associated with Childhood B-Cell Precursor Acute Lymphoblastic Leukemia: From Biological Significance to Precision Medicine Opportunities

**DOI:** 10.3390/biomedicines13071626

**Published:** 2025-07-02

**Authors:** Daniel Martínez Anaya, Johana Itzel Rodriguez Ruiz, María del Pilar Navarrete-Meneses, Patricia Pérez-Vera

**Affiliations:** 1 Laboratorio de Genética y Cáncer, Instituto Nacional de Pediatría, Mexico City 04530, Mexico; alejandro.bqd@gmail.com (D.M.A.); johanairr24@gmail.com (J.I.R.R.); peachnavarrete@hotmail.com (M.d.P.N.-M.); 2 Posgrado en Ciencias Biológicas, Universidad Nacional Autónoma de México, Mexico City 04510, Mexico; 3 Becaria de la Dirección General de Calidad y Educación en Salud, Secretaría de Salud, Mexico City 11400, Mexico

**Keywords:** B-cell precursor acute lymphoblastic leukemia, pathogenic variants, genetic biomarkers, germline predisposition, precision medicine

## Abstract

B-cell precursor acute lymphoblastic leukemia (B-ALL) is characterized by a constellation of somatic pathogenic variants associated with malignant transformation. These variants have implications for clinical management by providing clinical biomarkers. Most B-ALL cases have a sporadic presentation. However, some patients may present the disease as the neoplastic manifestation of cancer predisposition syndromes caused by germline pathogenic variants. In these cases, genetic counseling and personalized oncologic management is mandatory, considering the patient’s sensitivity to conventional therapies. In this review, we have summarized current knowledge on the biological role and clinical relevance of somatic and germline pathogenic variants associated with B-ALL, and discuss three aspects of their application as biomarkers: (1) their usefulness to determine specific molecular subtypes, predicting prognosis and response to specific therapies, (2) their influence in genetic counseling and therapy adaptation for B-ALL in the context of underlying cancer predisposition syndromes, and (3) their detection and interpretation through methodologies. We also included a brief discussion on the need to reclassify variants of uncertain significance to clarify their clinical relevance. Finally, we discuss cases illustrating the impact of somatic and germline pathogenic variants in personalized medicine.

## 1. Introduction

Acute lymphoblastic leukemia (ALL) is an uncontrolled proliferation of abnormal lymphoid cells and their precursors that invade the bone marrow and extramedullary tissues [1]. In 2019, approximately 5930 individuals were diagnosed with ALL in the US, and nearly 1500 individuals died because of the disease [1]. This neoplasm is the most common childhood cancer, and approximately ~85% of cases involve the B-lymphoid lineage (B-ALL) [2]. Over the past 60 years, the overall survival rate for pediatric ALL in high-income countries has increased from ~10% in the 1960s to >90% at present, making this disease one of the most curable malignancies [3]. This success is attributed to factors such as the monitoring of minimal residual disease (MRD) after treatment. Additionally, the progress achieved in the discovery of the genomic landscape of B-ALL has substantially improved the survival rate for this disease. This progress has enabled the development of personalized therapeutic strategies that consider genetic biomarkers in diagnosis, prognosis, and therapeutic decisions [3,4,5].

Diagnosis of B-ALL traditionally begins with bone marrow cytomorphologic analysis and determination of cell lineage markers by immunophenotyping. Subsequent cytogenetic and molecular analysis allows the identification of prognostic biomarkers [6,7]. At the time of diagnosis, patients are stratified into clinical risk groups, which allows prediction of outcome and tailoring of treatment intensity [6,7].

According to the NCI criteria, high-risk (HR) patients are infants, children, or adolescents, of Hispanic, Native American, or Black ethnicities, presenting a white blood cell (WBC) count >50 × 10^9^/L, and harboring genetic biomarkers associated with poor prognosis. In contrast, standard risk (SR) patients are children aged 1–9 years of Caucasian or Asian ethnicity who typically have a WBC count <50 × 10^9^/L, and have genetic biomarkers associated with favorable prognosis [6,7].

Chemotherapy regimens typically comprise 2 to 2.5 years and include three phases: A 4–6-week remission induction therapy aimed to destroy as many leukemic blasts as possible to restore normal hematopoiesis; this phase includes the use of glucocorticoids, vincristine, asparaginase, and intrathecal therapy. In general, this is the baseline chemotherapy for SR patients, while HR patients require higher doses of antileukemic drugs. The consolidation phase (6–8 months) includes high doses of methotrexate to prevent relapses. Finally, the maintenance phase (18–30 months) includes the use of antimetabolites [6,7].

The assessment of early response to treatment by measuring MRD allows prediction of relapse and adjustment of the chemotherapy intensity. A high MRD level at the end of the remission induction phase is a criterion for increasing the intensity of treatment or even considering hematopoietic stem cell transplantation (HSCT). MRD levels can be measured with high sensitivity using next-generation sequencing (NGS), which allows the detection of the disease by revealing the presence of genetic variants associated with leukemic cell surveillance [4,7].

## 2. The Role of Genetic Variants in the Development of B-ALL

**Figure 1:**
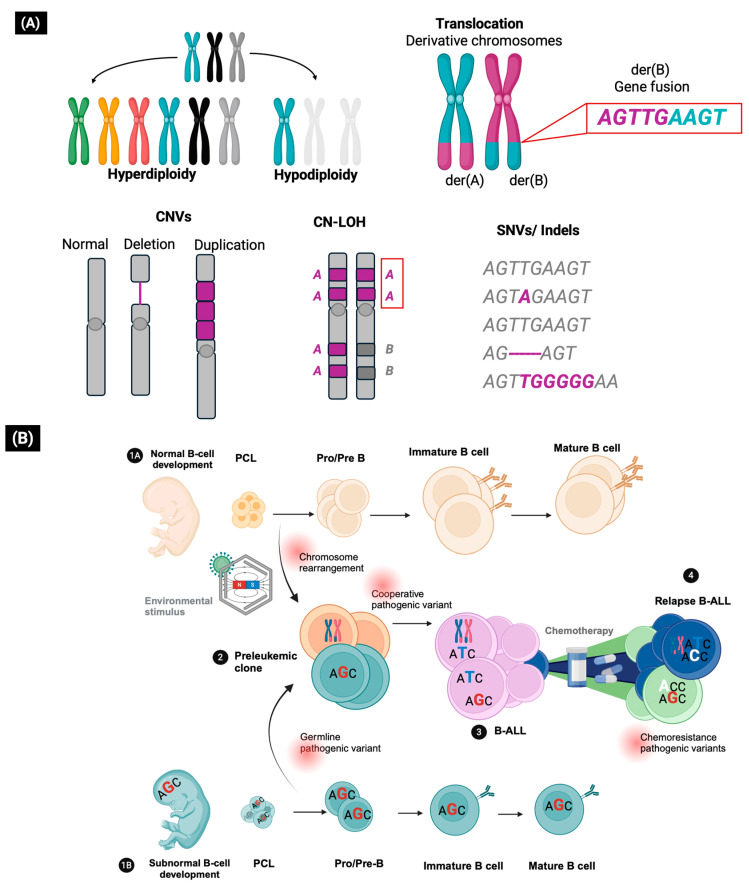
Genetic alterations involved in the pathogenesis of B-ALL. (**A**) Types of genetic alterations. Gain and loss of whole chromosomes, translocations producing derivative chromosomes (der) carrying gene fusions (red box). Copy number alterations (CNVs), copy neutral loss of homozygosity regions (CN-LOH) (red box), single nucleotide variants (SNVs), and insertions and deletions at the sequence level (Indels). (**B**) Development of B-ALL. 1A: Normal lymphopoiesis. 1B: Germline predisposition can lead to subnormal lymphopoiesis with reduced progenitor cell populations. 2: The preleukemic clone has chromosomal rearrangements induced by environmental stimuli or by the effect of germline pathogenic variants. 3: The preleukemic clone is susceptible to acquiring cooperative variants that drive its transformation to B-ALL. 4: Chemotherapy may promote the emergence of chemoresistance variants in new or pre-existing clones that drive relapse.

B-ALL is driven by a constellation of genetic lesions, which are mainly somatic changes that can be divided into gross chromosomal alterations and cryptic variants [5]. Chromosomal alterations are recurrent in B-ALL and can be detected by conventional analysis techniques such as karyotype, FISH or RT-PCR due to their size and low variability. These alterations include gains (hyperdiploidy) or losses (hypodiploidy) of whole chromosomes and translocations that form fusion genes [5,8,9]. In contrast, cryptic variants (<5 Mb) include copy number variants (CNVs), which are duplications or deletions of large DNA segments, copy neutral loss of heterozygosity (CN-LOH) regions, gene fusions, DNA sequence insertions or deletions (Indels), and single nucleotide variants (SNVs) (Figure 1A). Due to their size and molecular heterogeneity, cryptic variants require the use of genomic techniques for their detection [4,10,11]. The genetic variants are classified according to their clinical significance, with pathogenic variants being those that show the strongest association with tumor initiation, progression, and survival [11].

**Figure 2:**
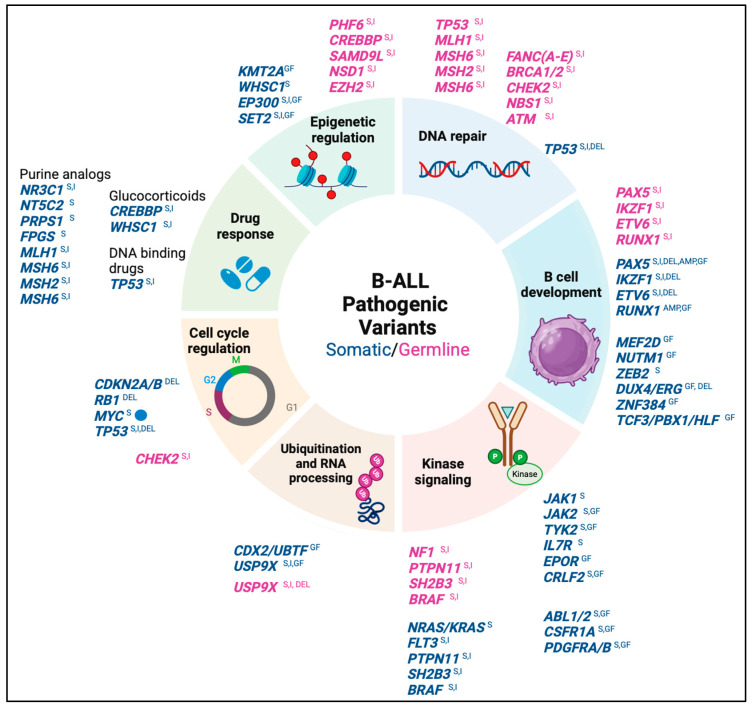
Genes altered by somatic and germline pathogenic variants and their molecular function. S: SNV variant. I: Indel variant. DEL: CNV deletion. AMP: Amplification. GF: Gene fusion.

Most B-ALL cases arise sporadically due to somatic chromosomal alterations that may occur during the early stages of embryonic life due to aberrant recombinase enzyme activity (triggered by environmental stimuli), promoting the development of a pre-leukemic clone that already has an initial predisposition to malignant transformation (Figure 1B) [12,13,14]. Chromosomal alterations are considered initial events in leukemogenesis, whereas pathogenic somatic variants are considered cooperative events that allow disease progression through the involvement of oncogenes and tumor suppressor genes relevant for lymphoid development, cell cycle regulation, kinase signaling, chromatin structure regulation, and drug response (Figure 2) [5,15,16].

Some cases of B-ALL are caused by genetic predisposition, such as some constitutional chromosomal abnormalities, e.g., trisomy 21, which is associated with a high risk of B-ALL. In contrast, common low-penetrance germline variants typically affect non-coding regions of genes involved in the normal function of lymphoid progenitors. Individually, these variants have a modest effect, conferring a one- to two-fold increase in the relative risk of developing the disease, although their co-occurrence in an individual can increase the risk of leukemia by up to nine-fold [12,17]. In contrast, rare pathogenic germline variants are highly penetrant and confer a >10-fold increased risk of developing leukemia. These variants commonly involve coding regions of genes encoding transcription factors involved in lymphoid differentiation, proteins associated with DNA damage response, and regulators of signaling pathways involved in cell proliferation (Figure 2) [12,18,19].

There is a complex interplay between somatic and germline genetic variants in the biology of B-ALL. Germline pathogenic variants predispose to the development of B-ALL by promoting subnormal lymphopoiesis in which lymphoid progenitors are susceptible to the acquisition of somatic pathogenic variants (Figure 1B). In another scenario, germline pathogenic variants may lead to epigenomic disruptions that increase the likelihood of the acquisition of cooperative variants, driving the transformation of pre-leukemic clones [19,20].

## 3. The Somatic Pathogenic Variants as Clinical Biomarkers for B-ALL

Based on the AMP/ASCO/CAP and ACMG/CGC 2017–2019 guidelines, pathogenic variants are classified into two levels according to their ability to serve as biomarkers for diagnosis, prognosis, or response to specific therapy [10,11,16,21].

- Tier I (with strong clinical significance): These variants constitute the molecular taxonomy of B-ALL and produce a biochemical or immunological phenotype associated with specific gene expression profiles, thus, allowing the identification of molecular subtypes with prognostic value [2,22]. Some of these variants have therapeutic value because they allow the prediction of FDA-approved therapies in pediatric patients with B-ALL. Based on the level of evidence, tier 1A variants are described in the World Health Organization (WHO) 2022 classification of lymphoid tumors and/or in professional clinical management guidelines for B-ALL (e.g., COG guidelines), while level 1B variants are recognized by consensus of disease experts based on multiple studies from different groups [10,11].
- Tier II variants (with potential clinical significance): These variants are recurrent in different hematological and non-hematological neoplasms, and some of them may integrate a profile of alterations that, in coexistence, have prognostic value. The presence of tier 2 variants may explain the heterogeneity in clinical response among patients with the same molecular subtype of B-ALL [10,11,16,22].

The prevalence of genetic somatic biomarkers with prognostic value is influenced by factors such as age, ethnicity, and clinical risk group [2]. In general, 70% of B-ALL cases harbor some of the sentinel chromosomal alterations defining the classical molecular subtypes, the remaining cases are identified as B-other and are characterized by a diversity of pathogenic variants defining new molecular subtypes or novel provisional entities. [23,24].

To increase the accuracy of clinical risk stratification, the identification of biomarkers with prognostic value needs to be performed in the early stages of the disease, because patients with unfavorable or intermediate prognostic biomarkers could benefit from increased chemotherapy intensity and/or treatment with drugs against targetable pathogenic variants. Conversely, patients with favorable prognostic biomarkers could benefit from reduced chemotherapy intensity if they show an early response with negative MRD levels [2,4,16,22,25].

### 3.1. Biomarkers Associated with Poor Prognosis

**Figure 3:**
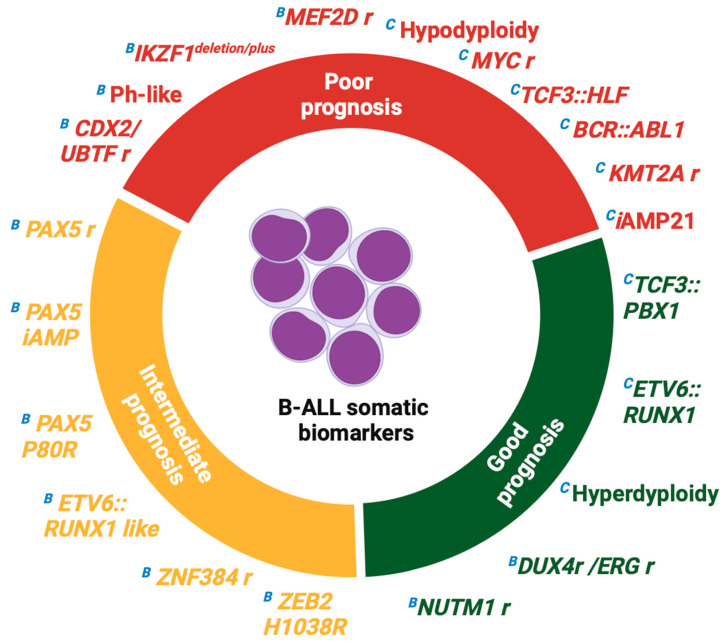
Somatic biomarkers with clinical value in B-ALL. They are grouped according to their prognostic value. C: Classic biomarkers detected by conventional analysis techniques. B: Biomarkers within the B-other group and detected only by genomic analysis techniques.

In this section, we describe some features of known and newly described somatic biomarkers associated with poor prognosis in B-ALL (Figure 3 and Table 1):

**Table 1:** Somatic Pathogenic Variants Recognized as Clinical Biomarkers of B-ALL.

B-ALL Molecular Subtypes/Profiles	AMP Classification †	General Frequency	Detection Technique	Common Underlying Pathogenic Variants	Therapeutic Approaches	References
POOR PROGNOSIS						
Hypodiploidy (Patterns of multiple chromosome losses)	Tier 1A Dx/Px	1–2%	Karyotype, FISH, CMA, WGS RNA-seq^GEP^	*TP53* germline variants	Intensive chemotherapy and HSCT	[2,25,26,27]
*TCF3::HLF* fusion	Tier 1A Dx/Px	<1%	Karyotype, FISH RT-PCR, WGS, RNA-seq ^GF/GEP^	*PAX5, BTG1*, *CDKN2A/B* deletions and RAS signaling activating variants	Venetoclax § NCT05292664 Intensive chemotherapy	[27,28,29]
*MYC* rearrangement *MYC* juxtaposed with *IGH, IGK, IGL*	Tier 2	<1%	Karyotype FISH WGS RNA- seq^GF/GEP^	*CDKN2A/B*, *RB1* deletions RAS signaling activating variants	Mature B-cell leukemia/lymphoma chemotherapeutic regimen	[27,30]
*BCR::ABL1* fusion	Tier 1A Dx/Px/Tx	2–4%	Karyotype, FISH, RT-PCR WGS, RNA-seq^GF/GEP^	*IKZF1* deletion	Intensive chemotherapy and Imatinib mesylate	[26,27]
Ph-like	SNVs, CNVs and GFs involving kinase genes	10–15%	CMA WGS RNA-seq	*IKZF1* deletions	Intensive chemotherapy and tyrosine kinase inhibitors	[22,27]
Ph-like positive to *CRLF2* rearrangement or mutation (See supplementary Table S1)	Tier 1A Dx/Px/Tx	~50% Ph-like	FISH^rearrangement^ CMA *^P2RY8::CRLF2^* WGS^rearrangement, PM^ RNA-seq ^GF/PM/GEP.^	*IKZF1* deletions and RAS/JAK-STAT signaling activating variants	Intensive chemotherapy and Ruxolitinib (JAK inhibitor) §NCT02723994 §NCT04996160 §NCT03117751	[3,27,28,31]
Ph-like positive to JAK-STAT class fusions (See supplementary Table S1)	Tier 1A Dx/Px/Tx	~7–10% Ph-like	WGS^rearrangements^ RNA- seq^GF^ FISH^rearrangements^	Deletions in lymphoid differentiation genes. RAS signaling activating variants.	Intensive chemotherapy and Ruxolitinib §NCT02723994 §NCT04996160 §NCT03117751	[3,22,28,31]
Ph-like positive to ABL class fusions (See supplementary Table S1)	Tier 1A Dx/Px/Tx	~12% Ph-like	WGS^rearrangements^ FISH^rearrangements^ CMA *_NUP214::ABL1_* RNA- seq^GF^	Deletions in lymphoid differentiation genes RAS signaling activating variants	Intensive chemotherapy and Dasatinib (Abl/Src inhibitor) §NCT02883049 §NCT03117751 §NCT03020030	[3,22,28,31]
Ph-like positive to JAK-STAT and RAS activating point mutations	Tier 2	~6–11% Ph-like	WGS^PM^ WES^PM^ RNA- seq^PM^	Deletions in lymphoid differentiation genes	Intensive chemotherapy and Ruxolitinib § NCT02723994 § NCT04996160 § NCT03117751	[3,22,28,31]
Ph-like positive to other kinase fusions (See supplementary Table S1)	Tier 1A Dx/Px/ Tx (*NTRK3* fusion)	~1% Ph-like	WGS, FISH RNA- seq^GF^	Deletions in lymphoid differentiation genes RAS signaling activating variants	Intensive chemotherapy and *NTRK3* inhibitors §NCT03066661 Entrectinib § NCT03834961 Larotrectinib	[3,22,28,31]
iAMP21 (21q22.12 chromotripsis with multiple *RUNX1* copies)	Tier 1A Dx/Px	~2%	Karyotype, FISH CMA, WGS, RNA-seq^GEP^	*SH2B3* loss of function variants	Intensive chemotherapy	[27,32]
*KMT2A* rearrangements	Tier 1A Dx/Px	1–2%	Karyotype, FISH, RT-PCR, WGS, RNA-seq^GF/GEP^	Subclonal RAS signaling activating variants	Azacitidine and Histone Methyltransferase inhibitors § NCT03724084	[27,33]
*MEF2D* rearrangement (See supplementary Table S2)	Tier 1B Dx/Px	~4–5%	FISH WGS RNA- seq ^GF/GEP^	Loss of function variants in *PHF6*	HDACs Vorinostat Quisinostat (preclinical evidence)	[27,34,35]
*CDX2/UBTF* rearrangements	Tier 1B Dx/Px	<1%	WGS FISH RNA- seq^GF/GEP^	*ETV6* monoallelic deletion *PAX5::ZCCHC7* estructural variant	Intensive chemotherapy	[27,36,37,38]
*IKZF1* c.475A>T p. N159Y *IKZF1* Deletion *IKZF1*^plus^ Profile	Tier 1B Dx/Px Tier 1B Px (*IKZF1* deletions) Tier 2 Px (IKZF1 ^plus^ Concomitant deletions)	<1% ~15% ~6%	WGS, RNA- seq^PM/GEP^ WGS CMA FISH	Unknown *IKZF1* whole or intragenic deletion are underlying lesions in various B-ALL molecular subtypes Concomintant deletions in *PAX5*, *CDKN2A*, *CDKN2B* (homozygous) or *PAR1* in absence of *ERG* deletion	Focal adhesion kinase inhibitors and rethinoids (preclinical evidence)	[27,39]
GOOD PROGNOSIS						
**Hyperdiploidy** **(Patterns of multiple chromosome gains)**	Tier 1A Dx/Px	20–30%	Karyotype FISH CMA WGS RNA-seq ^GEP^	RAS signaling activating variants	Antimetabolite based chemotherapy	[9,22,40]
*TCF3::PBX1* fusion	Tier 1A Dx/Px	2–6%	Karyotype FISH RT-PCR WGS RNA-seq ^GF/GEP^	*PAX5*, *CTCF* and *SET2* amplifications. *CDKN2A* deletions	Intensive chemotherapy	[27,41]
*ETV6::RUNX1* fusion	Tier 1A Dx/Px	15–25%	Karyotype FISH RT-PCR WGS RNA-seq ^GF/GEP^	*PAX5* deletions and *WHSC1* PM	Standard chemotherapy	[27,40]
*DUX4/ERG* rearrangements	Tier 1B Dx/Px	4–6%	WGS FISH RNA-seq ^GEP^	*ERG* and *IKZF1* deletions	Intensive chemotherapy	[22,27,42,43,44]
*NUTM1* rearrangement (See supplementary Table S2)	Tier 1B Dx/Px	<2%	WGS FISH RNA-seq ^GF/GEP^	Deletions in lymphoid differentiation genes	Standard chemotherapy	[27,45]
INTERMEDIATE PROGNOSIS						
*PAX5* altered *PAX5* fusions (See supplementary Table S2) PAX5 intragenic amplification (iAMP) *PAX5* c.239C>G p.Pro80Arg	Tier 1B Dx/Px	3.3–9.3% for PAX5 fusions or iAMP 0.4–1.9% for P80R mutation	WGS CMA FISH RNA-seq ^GF/PM/GEP^	*CDKN2A/B* deletions RAS/JAK-STAT signaling activating variants.	Intensive chemotherapy	[15]
*ETV6::RUNX1* like (See supplementary Table S2)	Tier 1B Dx/Px	<5%	WGS FISH RNA-seq^GEP/GF^	*ETV6, IKZF1 and ARPP21* deletions *ETV6* germline variants	Intensive chemotherapy	[27,42]
*ZNF384* rearrangement (See supplementary Table S2)	Tier 1B Dx/Px	1–6%	WGS WES FISH RNA-seq^GEP/GF^	Loss of function variants in *CREBBP, EP300, KDM6A*, *CHD4* and *CHD8* epigenetic regulators RAS signaling activating variants	Intensive chemotherapy	[27,46,47,48]
*ZEB2* mutated and *IGH::CEBPE* fusion	Tier 1B Dx/Px	<1%	WGS, WES, RNA-seq ^GEP/PM^	RAS signaling activating variants	Undefined	[27]

† Classification based on AMP/ASCO/CAP 2019 guidelines. Dx: Diagnostic value. Px: Prognostic value. Tx: Therapeutic value. HSCT: Hematopoietic stem cell transplantation. WGS: Whole genome sequencing. WES: Whole exome sequencing. CMA: Chromosome microarray analysis. FISH: Fluorescence in situ Hybridization. RNA seq: RNA sequencing. GF: Gene fusion. PM: Point mutation. GEP: Gene expression profile. § Clinical trial ID available on https://clinicaltrials.ucsf.edu or https://clinicaltrials.gov accessed on 18 February 2025.

Hypodiploidy: At the cytogenetic level, this subtype is characterized by a karyotype with fewer than 44 chromosomes, with patients with near-haploid karyotypes (24–31 chromosomes) and low hypodiploidy (32–39 chromosomes) having the worst prognosis. At the molecular level, most cases harbor pathogenic variants in *TP53* that are also present in non-tumor cells, suggesting a germline etiology and the manifestation of B-ALL as part of the neoplastic spectrum of Li-Fraumeni syndrome [2,25,26]. This subtype is associated with a poor prognosis and low event-free survival (EFS) rates (<38.3%), particularly in patients with a positive MRD at the end of induction. Most patients are treated with HSCT once remission is achieved, although the efficacy of this intervention and its potential impact on patient prognosis remains to be elucidated.

t(17;19)(q22;p13): This translocation is very rare in pediatric B-ALL, and is associated with a poor prognosis with EFS <2 years. At the molecular level, the translocation generates the *TCF3::HLF* gene fusion on derivative chromosome 19, which is an initial event in leukemogenesis and is often accompanied by CNVs in lymphoid differentiation genes and activating variants of RAS signaling present in subclones. Cells carrying the *TCF3::HLF* gene are resistant to conventional chemotherapy but show sensitivity to Venetoclax, an inhibitor of the apoptosis regulator BCL-2 [9,29].

*MYC* rearrangements: This is a very rare subtype in pediatric B-ALL and is associated with an unfavorable prognosis. It is characterized by chromosomal translocations that cause arrest of lymphoid differentiation by promoting the overexpression of the *MYC* gene through its repositioning toward immunoglobulin chain gene clusters. *MYC* rearranged lymphoblasts show FAB L2 or L3 cytomorphology without surface expression of immunoglobulin light chains and can be confused with mature B-cell leukemia/lymphoma; nevertheless, the absence of somatic hypermutation (VDJ rearrangement of the *IGVH* genes) in pediatric B-ALL is the principal difference [27,30].

t(9;22) (q34;12) and Ph-like subtypes: The t(9;22) originates the Philadelphia chromosome (derivative chromosome 22), which carries the *BCR::ABL1* oncogene, encoding a chimeric kinase that deregulates ABL-class signaling. This subtype confers a poor prognosis, but the addition of Imatinib mesylate to intensive chemotherapy significantly improves prognosis [25,26]. Similarly, Ph-like patients have high-risk clinical features such as elevated WBC counts, higher prevalence in adolescents and young adults, suboptimal response to remission induction chemotherapy, high relapse rates, and poor overall survival [25,26].

At the molecular level, the Ph-like subtype possesses a transcriptome similar to the Philadelphia-positive subtype but lacks the t(9;22) chromosome translocation and shows a plethora of pathogenic variants that deregulate the activity of lymphoid developmental transcription factors (mainly deletions of *IKZF1*), cytokine receptors, and tyrosine kinase signaling pathways. In general, *CRLF2* rearrangements are the most common abnormality, occurring in approximately 50% of Ph-like cases. The *CRLF2* gene encodes a transmembrane receptor that forms a heterodimer with the alpha chain of the interleukin-7 receptor in the presence of thymic stromal lymphopoietin ligand. This gene can be altered by deletions, translocations, and point mutations that deregulate JAK-STAT signaling (Supplementary Figure S1A), half of these cases harbor activating pathogenic variants in the *JAK1* and *JAK2* kinases, which are important mediators of hematopoietic signaling. In this context, it is important to identify the JAK2 R683G mutation in patients with *CRLF2* abnormalities because it confers resistance to a panel of JAK inhibitors, including Ruxolitinib [49]. Other relevant pathogenic variants include ABL or JAK-STAT class gene fusions (Supplementary Figure S2A), activating variants of the RAS signaling pathway, and gene fusions leading to deregulation of other kinase signaling pathways (Supplementary Figure S3A) [25,31,50].

Kinase dysregulation can be successfully treated with tyrosine kinase inhibitors (TKIs) such as Imatinib and Dasatinib, which abolish the ABL-class signaling, or with Ruxolitinib, which inhibits the JAK-STAT class signaling. The efficacy of TKIs has been demonstrated in cell lines and xenograft models of B-ALL. Currently, there are reports of Ph-like patients showing good responses to the use of TKIs in combination with conventional chemotherapy. These clinical and experimental observations have prompted the development of clinical protocols focused on determining the efficacy and safety of TKIs for the treatment of pediatric Ph-like B-ALL [31,50].

Intrachromosomal amplification of chromosome 21 (iAMP21): This subtype is characterized by chromothripsis (multiple cycles of amplification through DNA cleavage and splicing in contiguous regions) of chromosome 21. Cells carrying iAMP21 have more than 3 copies of the *RUNX1* gene per cell [51]. These patients typically present low to moderate WBC counts and a high relapse rate following standard risk-adjusted chemotherapy. Nevertheless, the risk of relapse can be reduced to less than 20% with high-risk adapted chemotherapy [51,52].

*KMT2A* rearrangement: At the molecular level, this subtype is defined by the presence of translocations at 11q23 loci, that fuse the 5′ region of the *KMT2A* gene (which contains the methyltransferase domain) with the 3′ region of a variety of gene partners. The leukemic cells exhibit a mixed lineage immunophenotype with the co-expression of lymphoid (CD34 and CD19) and myeloid (CD15 and CD65) markers. *KMT2A* rearranged B-ALL is characterized by a high incidence in children under 1 year of age and a poor prognosis with high WBC counts and leukemic extramedullary infiltration. Treatment consists of intensive chemotherapy and allogeneic HSCT; although most children achieve remission (82.5%), this is usually short-lived, with rapid relapse and poor survival. Other therapeutic options based on histone deacetylase inhibitors (HDACi) in combination with standard chemotherapy are under preclinical investigation [33].

*MEF2D* rearrangement: The *MEF2D* gene encodes a transcription factor required for lymphoid development and can form gene fusions with several partners, including *BCL9*, which is the most common. All fusions retain the DNA-binding domain, resulting in deregulation of *MEF2D* target genes (except for *MEFD2::CSF1R*, which belongs to the ABL-class Ph-like subtype). Cells positive to *MEF2D* fusion have an abnormal CD10-, CD38+ immunophenotype and are characterized by highly vacuolated basophilic nuclei like the mature B-cell leukemia morphology. This B-ALL molecular subtype has few cooperative lesions, and the most common are pathogenic SNVs in the transcriptional regulator *PHF6* (50% of cases). Clinically, it is a biomarker of poor prognosis with low EFS rates, but similar to *KMT2A* rearranged B-ALL, it also shows overexpression of the *HDAC9* gene and, thus, is responsive to HDACi therapy [27,34].

*CDX2/UBTF* rearrangement: This recently discovered B-ALL subtype is characterized by the co-occurrence of two alterations, a ~280 Kb microdeletion that relocates the *CDX2* gene next to the *FLT3/PAN3* gene enhancer, causing its overexpression, and a ~10 Kb microdeletion at 17q21.31 that results in the formation of *UBTF::ATXN7L3* gene fusion (Supplementary Figure S2A). *CDX2* regulates homeotic gene expression during embryonic hematopoiesis, and *UBTF* is a nuclear protein that regulates rRNA transcription. The biological significance of these alterations is still unknown; however, both produce a CD10-, IgM+ immunophenotype and a specific gene expression profile. This is a rare subtype in pediatric B-ALL, with most cases occurring in adolescents and young adults with a female preponderance. It confers an unfavorable prognosis with a high relapse rate and treatment refractory with high MRD levels. Treatment with intensive chemotherapy improves the prognosis in adults, although the response in pediatric patients remains suboptimal [36].

Somatic pathogenic variants in *IKZF1*: *IKZF1* is located at 7p12.2 and encodes the Ikaros protein, a transcription factor that plays a key role in lymphoid differentiation. Ikaros has six zinc fingers, four of which are in the N-terminal domain and allow its binding to DNA, and two of which are in the C-terminal domain and allow its homo- or heterodimerization (Supplementary Figure S2B). The most common alterations of *IKZF1* in B-ALL are monoallelic deletions of the whole gene, with pathogenic effect by haploinsufficiency. Another common alteration is the intragenic deletion from exons 4 to 7, resulting in the Ik6 isoform. This isoform loses the DNA binding domains and retains the dimerization domains, allowing it to exert a negative dominant effect on wild-type transcripts, reducing the function of genes that regulate the self-renewal capacity of lymphoid progenitors [53,54].

*IKZF1* deletions (*IKZF1^del^*) confer an unfavorable prognosis, according to the DFCI ALL consortium study 05-001, *IKZF1^del^* [53]. *IKZF1^del^* is commonly an underlying lesion in poor-prognosis B-ALL molecular subtypes, such as the Ph+ and Ph-like cases, in which it is highly prevalent (70% and 68%, respectively). In contrast, favorable prognostic subtypes such as hyperdiploidy and t(12;21) show a lower prevalence (15% and 3%, respectively), although even in t(12;21) cases, *IKZF1^del^* reduces 5-year EFS, reinforcing its negative impact [54].

The prognostic value of *IKZF1^del^* is modified by the epistatic effect of CNVs in other lymphoid development genes such as *ERG*. The coexistence of *IKZF1* and *ERG* deletions reduces the negative prognostic impact. In contrast, the absence of the *ERG* deletion together with positive deletions in the *CDKN2A/B*, *PAX5*, and *PAR1* genes constitutes the *IKZF1^plus^* profile, which amplifies the negative impact of *IKZF1del* in patients with MRD > 10^−^^4^ at the end of induction therapy, resulting in a 5-year EFS of 53%, 79%, and 89% in the *IKZF1^plus^*, *IKZF1^del^*, and normal *IKZF1* patients, respectively [54,55].

The heterozygous variant IKZF1p.Asn159Tyr defines a subtype of B-ALL with a specific gene expression profile. This subtype is rare, and its molecular features and prognostic value are currently unknown. The Asn159 residue, which is in the DNA binding domain (Supplementary Figure S2B), disrupts the function of IKZF1, promoting its aberrant nuclear localization and increasing cellular adhesion. In contrast to other subtypes with underlying *IKZF1* alterations, the N159Y variant increases the overexpression of chromatin remodelers and regulators of kinase signaling that are not deregulated in *IKZF1^del^* subtypes [48].

### 3.2. Biomarkers Associated with Good Prognosis

In this section, we describe some features of known and newly described somatic biomarkers associated with good prognosis in B-ALL (Figure 3 and Table 1):

Hyperdiploidy: This is the most common alteration in B-ALL and is characterized by a karyotype with 51–67 chromosomes. Trisomies of chromosomes X, 4, 6, 10, 14, 17, 18, X and tetrasomy 21 are the most common gains. Variants in genes involved in the RAS pathway and epigenetic regulation are common cooperating lesions in this subtype, but patients with hyperdiploidy generally have a favorable prognosis and are candidates for reduction of chemotherapy intensity [2,40].

t(1;19) (q23;p13.3): This translocation produces a derivative chromosome 19 carrying the oncogenic fusion *TCF3::PBX1*, which encodes a transcription factor that confers self-renewal capacity and disrupts lymphoid differentiation. Initially, this subtype was considered to have an intermediate prognosis, attributable to its propensity for central nervous system relapse. However, subsequent research has demonstrated that intensive chemotherapy treatments can improve the prognosis and, thus, it is now considered to have a favorable prognosis [9,26].

t(12;21) (p13;q22): This translocation produces a derivative chromosome 12 carrying the oncogenic fusion *ETV6::RUNX1*, which requires cooperating alterations to induce leukemogenesis. It is generally associated with a favorable prognosis with good response to low-intensity chemotherapy [2,26].

*DUX4/ERG* rearrangement: This subtype is defined by a rearrangement that affects the *DUX4* gene, which encodes a homeodomain transcription factor involved in lymphoid differentiation and is not expressed in most somatic tissues. This gene is located within the D4Z4 repeat region in the subtelomeric region of chromosomes 4q and 10q, and its insertion into the *IGH* locus results in its overexpression. It can also be inserted into intron 6 of the *ERG* transcription factor, and this rearrangement also results in the expression of a non-canonical *ERG* transcript that contains a truncated C-terminal domain but retains its DNA-binding domains. This transcript has an aberrant activity that promotes deletion of the second allele of *ERG,* driving leukemic transformation (Supplementary Figure S2C). *ERG* and *IKZF1* deletions are frequent secondary events in patients with *DUX4* rearrangement (50–63% and 40%, respectively). This subtype is more prevalent in adolescents and young adults. In pediatric patients, it presents at a median age of 6.5 years with low WBC counts and elevated MRD levels. There is a slow response to remission induction with standard therapy. However, the prognosis undergoes a substantial transformation, shifting from a poor prognosis to a favorable one, particularly in cases where intensive chemotherapy is applied [22,43,44].

*NUTM1* rearrangement: This is a rare entity that mainly affects infants and is associated with a good response to standard chemotherapy. The presence of *NUTM1* fusion with numerous partners is characteristic of this subtype, with *BRD9* being the most prevalent. All rearrangements retain almost the entire coding region of *NUTM1* and result in overexpression of this gene, although the associated biological effect is still unknown [45].

### 3.3. Biomarkers Associated with Intermediate Prognosis

In this section, we describe some features of known and newly described somatic biomarkers associated with intermediate prognosis in B-ALL (Figure 3 and Table 1):

*PAX5* abnormalities: *PAX5* is a tumor suppressor gene involved in the process of lymphoid differentiation. Two distinct somatic pathogenic variants in *PAX5* have been identified, each exhibiting significant prognostic value. These genetic abnormalities have been shown to promote leukemogenesis through the inhibition of transcriptional activity associated with *PAX5* haploinsufficiency. These variants are described as follows: (1) Rearrangements of *PAX5*. Include in-frame or out-of-frame gene fusions resulting in loss or disruption of the transactivation domain. The most common gene partners are *ETV6*, *NOL4L,* and *CBFA2T3* (fusions with *ZCCHC7* and *JAK2* belong to other subtypes). Another structural variant is the intragenic amplification of *PAX5* exons 2–5, which encode the DNA-binding domain and the octapeptide domain. Finally, mainly heterozygous and missense SNVs at residues Arg38 and Arg140 of the DNA binding domain (Supplementary Figure S2B), nonsense splice site variants, and reading frameshift Indels clustered in the distal region of the protein have been identified [15,22]. (2) PAX5 Pro80Arg: Unlike other mutations in *PAX5*, the p.Pro80Arg variant produces a specific gene expression profile, is present in the homozygous state due to the inactivation of the second allele (through deletion, loss of heterozygosity, or mutation) and impairs the activity of the DNA binding domain, causing arrest of lymphoid differentiation at the pre-proB stage [15,22].

*ETV6::RUNX1-like*: A molecular subtype defined by a CD27+ CD44 dim/negative immunophenotype and a gene expression profile similar to that of leukemic cells harboring the t(12;21), but without the *ETV6:: RUNX1* fusion. Instead, fusions of *ETV6* with partners other than *RUNX1* are observed, as well as concomitant deletions in the other allele of *ETV6* and *IKZF1* deletions. In addition, some cases carry germline pathogenic variants in *ETV6*. As *ETV6* and *IKZF1* are key genes for lymphoid differentiation, it is assumed that the combination of these alterations may disrupt the transcriptome in the same way as the *ETV6::RUNX1* fusion. This subtype is rare and restricted to pediatric patients with low WBC counts who achieve remission with intensive and standard-risk chemotherapy. Nonetheless, there have been reports of relapses, and research examining cohorts of patients who have received uniform treatment is still lacking. As a result, the prognosis remains uncertain [42,46,56].

*ZNF384* rearrangement: The *ZNF384* gene encodes a C2H2-type zinc finger protein that binds to the promoters of several genes involved in extracellular matrix production, JAK-STAT pathway activation, and lymphoid differentiation. This gene fuses with multiple partners (*TCF3*, *TAF15,* and *EP300* are the most common) and produces a specific gene expression profile and immunophenotype. The immunophenotype is characterized by low expression of CD10 and the presence of the myeloid markers CD13 and CD33. Loss-of-function variants in epigenetic regulatory genes and gain-of-function variants in RAS pathway genes are common co-occurring lesions in this subtype. Clinical features depend on the gene fusion, with the *EP300::ZNF384* and *TCF3::ZNF384* fusions commonly associated with high WBC counts, relapse, and presentation at a median age of 5 years, whereas the remaining fusions are more common in adolescents and are associated with favorable and high-risk clinical features. Therefore, the prognosis associated with the specific subtype remains indeterminate [42,46,57].

*ZEB2* mutated/*IGH::CEBPE*: This subtype is defined by recurrent SNVs at residues His1038 and Gln1072 located in the DNA binding domain of the ZEB2 (Supplementary Figure S2B), which is a zinc finger homeotic protein involved in lymphoid differentiation. When this mutation co-occurs with the *IGH::CEBPE* rearrangement, a characteristic gene expression profile is produced. However, the role of both alterations in leukemogenesis is uncertain. *ZEB2* mutations may underlie other lesions, e.g., *DUX4* and *ZNF384* fusions. At the clinical level, *ZEB2* mutations occur in pediatric patients with a mean age of 8 years and standard WBC counts, these patients have a high relapse rate (50%) when treated with standard risk chemotherapy, which decreases in patients treated with high-risk chemotherapy (8%), suggesting that the prognosis depends significantly on the intensity of chemotherapy. Nevertheless, given their low frequency and association with other subtypes, the clinical relevance of *ZEB2* mutations remains to be elucidated [25,47,48].

### 3.4. Biomarkers Associated with Chemoresistance

Clinical risk stratification and appropriate intensification of therapy have led to a significant improvement in the prognosis of pediatric patients with B-ALL, although the risk of relapse remains high for some patients [3,40,57]. Response to chemotherapy depends on three main factors: (1) non-genetic variation (age, drug interactions, adherence). (2) The presence of common germline variants of low penetrance that modify the pharmacokinetics of antileukemic drugs, although their clinical value is controversial due to their low penetrance. (3) The presence of somatic pathogenic variants that may be present at diagnosis or acquired during chemotherapy treatment in specific leukemic clones that emerge during relapse and have a direct effect on the mechanism of action of antileukemic drugs [58,59].

Genomic analysis in patients before and after therapy has revealed the genetic landscape of relapsed B-ALL. Approximately 75% of pathogenic variants present at relapse are derived from pre-existing subclones that survive therapy and acquire cooperative mutations to become a relapse promoter clone (Figure 1B). These variants primarily involve RAS and JAK-STAT signaling genes that emerge at diagnosis as underlying pathogenic variants in multiple molecular subtypes of B-ALL. Some variants are specific or enriched in relapse clones, most commonly involving genes that regulate response to glucocorticoids (*NR3C1*, *CREBBP*, *WHSC1*), purine analogues and methotrexate (*MSH6*, *MSH2*, *PMS2*, *NT5C2*, *PRPS1* and *FPGS*), and DNA-binding antileukemic drugs (*TP53*) (Supplementary Figure S3) [60,61,62].

Variants in genes that regulate the response to glucocorticoids have primarily been found at relapse, but also at diagnosis. *NR3C1* mutations have been found in 0.9% of patients with newly diagnosed ALL and in 11.1–13% of patients with relapsed ALL. Loss-of-function mutations in *NR3C1* have been shown to influence glucocorticoid resistance [63]. *CREBBP* mutations have been detected in 18.3% of relapsed ALL cases. In contrast, mutations in this gene are uncommon in non-relapsed ALL [60]. *CREBBP* mutations are notably prevalent in patients with high hyperdiploid ALL [64]. *WHSC1* mutation frequency is 10% in standard-risk and high-risk ALL patients [63]. A study of 99 B-ALL cell lines harboring diverse gene fusions revealed that mutations in *NR3C1* tend to be more prevalent in cell lines with *MEF2D* fusions and *TCF3-HLF*. In cell lines harboring *CREBBP* mutations, *MEF2D* fusions were more prevalent, while *KMT2A* fusion was less prevalent. Regarding *WHSC1* mutations, *TCF3-PBX1* and *ETV6-RUNX1* incidences tended to be higher. Unfortunately, this study did not analyze hyperdiploid cell lines [65].

Variants in genes that respond to purine analogues are usually not detected at diagnosis and, thus, they are considered relapse-associated mutations that are possibly acquired during treatment within a minor subclone present at diagnosis [61]. *NT5C2* is particularly important as it is associated with mercaptopurine resistance and has an overall occurrence rate of 10% [66]. In general, the prevalence of mutations resulting from chemotherapy exposure in this group of genes is estimated to be 17% in patients with very early relapse (<9 months since diagnosis), 65% in early relapse groups (9–36 months) and 32% in late relapse groups (>36 months). Based on this, monitoring of chemoresistance variants during remission using high-throughput deep sequencing has been proposed [61].

Conversely, immunotherapy has transformed the treatment of relapsed B-ALL, exhibiting the advantage of not targeting the genetic alterations of the disease and the ability to overcome chemoresistance variants present in relapsed clones [67]. Blinatumomab is a monoclonal antibody that facilitates binding between T cells (CD3+) and B cells (CD19+), thereby promoting the destruction of malignant B cells. It has demonstrated improved efficacy compared to standard chemotherapy as a remission induction agent in patients with relapsed B-ALL, with moderate toxicity and an MRD-free response rate [67].

Therapy based on the use of chimeric antigen receptor T cells (CAR-T) has achieved 65–90% responses in clinical trials for the treatment of relapsed B-ALL. Phase I and II clinical trials have reported response rates of 50% at one-year post-treatment. This therapy represents the major therapeutic advance for B-ALL and may even replace HSCT. Based on the good response, this therapy has been approved by the FDA as Tisagenlecleucel [68,69,70].

## 4. Germline Pathogenic Variants as Biomarkers of Susceptibility to B-ALL

Germline predisposition to cancer has been recognized for decades, mainly in solid tumors [19]. Although most cases of pediatric B-ALL are sporadic, detailed genomic analyses in cohorts of patients with this disease have revealed that between 8.3 and 11% of cases carry SNV/Indel-type germline pathogenic variants in cancer predisposition genes [71,72,73]. Most of these variants follow an autosomal dominant (AD) inheritance pattern in which a single copy of the altered gene is sufficient to confer cancer risk, and the neoplastic phenotypes correlate with the gene expression pattern of the genes involved. Genetic variants expressed during the process of hematopoiesis have been identified as a contributing factor to the development of B-ALL, whereas variants in genes expressed in different cell lineages cause syndromes that predispose to solid tumors and sporadically to B-ALL [19,20].

### 4.1. Biomarkers Conferring High Susceptibility to B-ALL

**Figure 4:**
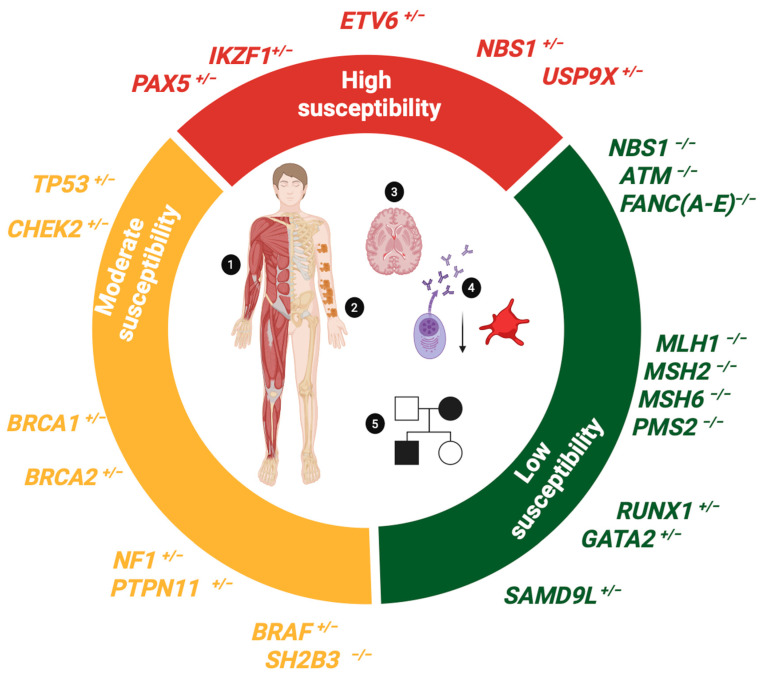
Genes affected by germline pathogenic variants grouped by B-ALL susceptibility. The clinical signs associated with genetic syndromes predisposing to B-ALL are shown. 1. Musculoskeletal disorders. 2. CALMS: Café au lait macules. 3. Neurological disorders. 4. Lymphopenia or thrombocytopenia. 5. Family history of neoplasms. Heterozygous genotype: *+/−*. Homozygous recessive genotype: *−/−*.

In this section, we describe constitutional entities that manifest B-ALL as the main neoplastic phenotype (Figure 4 and Table 2):

**Table 2:** Genetic Syndromes Associated with B-ALL Susceptibility.

Genetic Syndrome (OMIM #ID)	Gene (Loci)	Inheritance	Neoplastic Phenotype	Main Non-Neoplastic Phenotypic Characteristics	References
HIGH SUSCEPTIBILITY SYNDROMES					
*PAX5* Deficiency (615545)	*PAX5* (9p13.2)	AD	B-ALL	Immunodeficiency with low levels of B cells and immunoglobulins	[16,74,75]
*ETV6* Deficiency (616216)	*ETV6* (12p13.2)	AD	Hyperdiploid or *ETV6::RUNX1* like B-ALL	Thrombocytopenia	[56,76,77]
*IKZF1* Deficiency (613065)	*IKZF1* (7p12.2)	AD	B-ALL	Lymphopenia	[78]
*USP9X* Deficiency (300968)	*USP9X* (Xp11.4)	XLD	B-ALL restricted to female patients	Psychomotor delay and intellectual disability Congenital abnormalities	[79]
MODERATE SUSCEPTIBILITY SYNDROMES					
Li-Fraumeni syndrome (151623)	*TP53* (17p13.1)	AD	Breast cancer, sarcoma, glioblastoma, adrenal gland cancer Hypodiploid B-ALL	NA	[80,81,82]
Neurofibromatosis type 1 (162200)	*NF1* (17q11.2)	AD	Neurofibromas Gliomas, JMML B-ALL	CALMs Skeletal disorders Cognitive deficits	[83,84]
**Noonan/Leopard** **syndrome** **(163950/15110)** **Cardiofaciocutaneous syndrome** **(115150)** *SH2B3* Deficiency (NA)	*PTPN11* (12q24.13) (~50% cases) Other RAS genes *(SOS, BRAF1, RAF1, MAPK1*, *KRAS*, *NRAS)* *BRAF* (7q34) *SH2B3* (12q24.12)	AD AD AR	JMML Neuroblastoma Rhabdomyosarcoma Hyperdiploid B-ALL Non-Hodgkin’s lymphoma Hepatoblastoma Rhabdomyosarcoma B-ALL MPS/JMML B-ALL	Craniofacial Anomalies, Congenital Heart Anomalies CALMs Short stature Thrombocytopenia Hepatosplenomegaly Autoimmunity Hepatosplenomegaly Autoimmunity	[85,86,87]
LOW SUSCEPTIBILITY SYNDROMES					
Nijmegen syndrome (615545)	*NBN (NBS1)* (8q21.3)	AR	T-cell lymphoproliferative disorders B-ALL *	Craniofacial dysmorphias Developmental delay Immunodeficiency	[88,89]
**Constitutional mismatch repair deficiency syndrome.** (CMMRD) (619069)	*MLH1* (3p22.2) *MSH2* (2p21-p16.3) *MSH6* (2p16.3) *PMS2* (7p22.1)	AR	Colorectal cancer Glioblastoma T-cell lymphoproliferative disorders B-ALL *	CALMs Immunodeficiency	[90,91]
Fanconi anemia (Various genes)	Genes FANC(A-E) (Múltiple loci)	AR, XLR	AML Head and neck cancer B-ALL *	Dysmorphological syndrome CALMs Musculoskeletal disorders Intellectual disability	[92,93]
Ataxia telangiectasia syndrome (208900)	*ATM* (11q22.3)	AR	T-cell lymphoproliferative disorders B-ALL *	Neurological disorders Telangiectatic disorders Immunodeficiency	[94,95]
*RUNX1* Deficiency (601399)	*RUNX1* (21q22.12)	AD	MDS and T-ALL B-ALL *	Thrombocytopenia	[96]
*GATA2* Deficiency (614172)	*GATA2* (3q21.3)	AD	MDS, AML, JMML, T-ALL B-ALL *	Immunodeficiency	[97]
Ataxia pancytopenia Syndrome (159550)	*SAMD9L* (7q21.2)	AD	MDS, AML, B-ALL *	Neurological disorders Cytopenia Immunodeficiency	[98,99]

AD: Autosomal dominant. AR: Autosomal recessive, XLR: X-linked recessive. MPS: Myeloproliferative syndrome. MDS: Myelodysplastic syndrome. JMML: Juvenile myelomonocytic leukemia. NA: not available. CALMs: Café au lait macules. AML: Acute myeloid leukemia. * Some rare cases of B-ALL have been reported in patients with these genetic syndromes.

*PAX5* deficiency: The germline variants in *PAX5* show an AD inheritance pattern with incomplete penetrance; consequently, patients may be asymptomatic, develop immunodeficiency, or B-ALL. All reported variants are unique, except for *PAX5* c.113G > A p. Glu183Ser, which has been described in individuals from three unrelated families and involves a highly conserved residue of the octapeptide domain essential for normal gene function (Supplementary Figure S4). Complete loss of *PAX5* function is required for leukemogenesis, as all heterozygous carriers of germline variants in *PAX5* who develop B-ALL show somatic inactivation of the second allele [16,74]. According to the findings of functional studies, germline variants in *PAX5* have been demonstrated to exert a “hypomorphic” effect, characterized by a modest but significant reduction in transcriptional activity when compared to the normal protein. The conserved activity is sufficient to promote B-lymphocyte development but not to prevent leukemic transformation [74,75].

*ETV6* deficiency: This is an incompletely penetrant AD disorder characterized by thrombocytopenia and predisposition to ALL. It is caused by germline variants in *ETV6*, which encodes a transcriptional repressor essential for hematopoiesis. These variants have a frequency of <1% in patients with B-ALL, and approximately 23–30% of carriers develop the disease, mainly the hyperdiploid and *ETV6::RUNX1-like* subtype. Ninety-six individuals from 23 different families have been reported with recurrent variants involving conserved residues of the DNA-binding domain (Supplementary Figure S4); their functional effects include destabilization of DNA binding and protein delocalization with a dominant negative effect on the protein encoded by the normal allele [56,76,77].

*IKZF1* deficiency: Germline variants in *IKZF1* have an AD inheritance pattern with incomplete penetrance and can result in low levels of B cells or the development of B-ALL. Germline variants in *IKZF1* have a prevalence of 0.9% in pediatric patients with B-ALL and can occur in sporadic and familial cases. Unlike the *IKZF1* germline variants that cause the common variable immunodeficiency syndrome, the *IKZF1* B-ALL predisposition variants are located along the coding region of the gene (Supplementary Figure S4) and have a moderate to strong functional impact on the processes of dimerization, DNA binding, subcellular localization, cell adhesion, and drug response [78].

*USP9X* deficiency: Located on Xp11.4, *USP9X* is a tumor suppressor and oncogene that regulates the ubiquitylation of various cellular substrates. At the somatic level, it is altered by loss-of-function variants in 1.1% of B-ALL cases. This gene escapes X-chromosome inactivation and, when affected by germline pathogenic variants, causes embryonic lethality in males and X-linked mental retardation syndrome restricted to females. This is a rare dysmorphological syndrome with only 42 patients described in the literature, of which 7.1% developed pediatric B-ALL [79].

*NBS1* heterozygous variants: Carriers of homozygous pathogenic variants in the *NBS1* gene develop Nijmegen syndrome (NJS), an autosomal recessive disorder characterized by predisposition to lymphoid neoplasms, mainly of the T-cell type. In contrast, carriers of heterozygous variants in *NBS1* do not manifest NJS but are at risk of developing B-ALL [89]. *NBS1* encodes nibrin, which is a sensor of double-strand breaks in DNA and an adaptor for the activation of repair pathways for these lesions. The germline heterozygous NBN (c.657_661del5)p.K219fs variant was identified in 0.8% of a cohort of 4183 pediatric patients with B-ALL, a frequency similar to that of germline pathogenic variants in *PAX5*, *IKZF1*, and *ETV6*. Although most of these variants are of uncertain significance, functional studies have demonstrated a pathogenic effect mainly associated with alterations in protein stability. These variants show “hot spots” in the N-terminal domain at highly conserved residues (Supplementary Figure S4) and result in hypomorphic DNA damage response activity. However, the role of these alterations in the transformation of B-lymphoid precursors is still unclear [89,100,101].

### 4.2. Biomarkers Conferring Moderate Susceptibility to B-ALL

In this section, we describe constitutional entities that manifest solid tumors or myeloid malignancies as the predominant neoplastic phenotype, but occasionally, some carriers develop B-ALL (Figure 4 and Table 2):

*TP53* deficiency: *TP53* controls the response mechanisms to DNA damage. Its loss of function due to germline pathogenic variants results in Li-Fraumeni syndrome (LFS), which has an AD inheritance pattern and confers an almost 100% risk of developing cancer. The neoplastic spectrum of LFS is broad, with brain tumors being the most common and leukemias less frequent. Less than 1% of B-ALL cases are associated with the LFS syndrome, and most of these (65.4%) are characterized by a hypodiploid karyotype [81,82]. The germline pathogenic variants in *TP53* detected in LFS B-ALL patients are mainly clustered in the DNA binding domain (Supplementary Figure S4), and involve the Arg 248 residue in approximately 20% of cases [80,102,103]. Furthermore, some patients with pathogenic variants in genes that regulate p53 activity (e.g., *CHEK2*, *BRCA1*) show clinical criteria compatible with LFS [104].

Constitutional deficiency in RAS pathway genes: Germline loss-of-function variants in the *NF1* and *PTPN11* genes cause neurofibromatosis type 1 (NFS) and Noonan/Leopard syndrome (NLS), respectively. Both syndromes have AD inheritance and belong to a group of syndromes with constitutive dysregulation of RAS signaling known as RASopathies. They produce a phenotype characterized by body and skin malformations and a predisposition to brain tumors, myeloid leukemia, and occasionally B-ALL. The estimated frequency of NFS and NLS is ~0.12% and 0.02–0.04%, respectively, calculated in 4939 pediatric patients, predominantly with B-ALL and hyperdiploid karyotypes [80,105].

### 4.3. Biomarkers Conferring Low Susceptibility to B-ALL

In Table 2, we summarize cancer predisposition syndromes that mainly predispose to the development of solid tumors and myeloid and T-cell neoplasms in pediatric patients. These syndromes are associated with germline pathogenic variants in genes that occasionally cooperate in leukemogenesis; therefore, some rare cases of B-ALL have been reported [106].

### 4.4. Clinical Management of Patients with B-ALL Associated with Germline Predisposition

Pediatric patients with B-ALL should be screened for predisposing germline variants if they meet the following criteria (Figure 4) [106,107,108]:

- Consanguineous parents
- Presence of cytopenia in the patient and/or family members
- Dysmorphologic syndrome (musculoskeletal disorders)
- Pigmented lesions or other symptoms associated with RASopathies
- B-ALL of the hypodiploid or *ETV6::RUNX1-like* subtypes
- Family history of cancers included in the neoplastic spectrum of LFS
- Occurrence of a second primary neoplasm
- Siblings with childhood cancer or close relatives diagnosed with LFS spectrum cancers at age ≤ 45 years

Identification of the genetic syndrome underlying B-ALL is essential for the accurate clinical management of affected individuals and their families [107]. This requires a clinical evaluation by medical oncologists and geneticists that includes analysis of clinical and family history, physical examination for signs and symptoms associated with the genetic syndrome in question, and cytopathologic analysis for lymphopenia or thrombocytopenia. Once B-ALL susceptibility syndrome has been identified, it is strongly recommended that the patient be referred to a center with expertise in the management of hereditary cancer syndromes [107]. Clinical management of hereditary B-ALL should include identification of relatives who are carriers of the germline pathogenic variant and genetic counseling, selection of appropriate treatment considering the underlying genetic syndrome, and regular clinical surveillance to prevent the occurrence of second malignancies [107].

Treatment of patients with B-ALL associated with syndromes deficient in DNA repair mechanisms requires a delicate balance between therapeutic efficacy and toxicity (Table 2). Patients with LFS are highly sensitive to agents that directly damage DNA. Patients with NJS, ataxia-telangectasia and Fanconi anemia are particularly sensitive to radiation-based treatments. Patients with constitutional mismatch repair deficiency syndrome (CMMRD) are particularly sensitive to treatment with purine analogues. Currently, there is insufficient evidence to adjust chemotherapy in these patients; however, there are reports of patients who have benefited from low-sensitivity treatment [80].

Asymptomatic patients who carry germline pathogenic variants that predispose them to B-ALL should be monitored with complete blood counts at regular intervals, typically every 6–12 months. If indications of B-ALL emerge, the frequency of these counts should be repeated more frequently depending on the severity of the findings [106].

Cytogenetic and molecular analysis of the bone marrow should be considered to look for early signs of clonal evolution that may progress to B-ALL. The identification of dysplasia, increased lymphoblasts, cytogenetic and/or molecular abnormalities in the bone marrow requires consideration of preventive HSCT. Nevertheless, for patients who will receive allogeneic HSCT, the identification of relatives who are carriers of the germline pathogenic variant is critical to avoid selecting a donor who is a carrier of this variant [106].

## 5. Detection and Interpretation of Genetic Variants in B-ALL Patients

### Genomic Techniques Applied to B-ALL Analysis

Next-generation sequencing (NGS) refers to a set of advanced technologies that allow the analysis of the whole genome (WGS), exome (WES), or transcriptome (RNA-seq) in multiple samples simultaneously. These technologies have different applications in the characterization of variants in B-ALL. Depending on their depth and coverage, WES is useful for the detection of SNVs/Indels present in subclones, whereas WGS allows the detection of structural variants (translocations, inversions, insertions). RNA-seq enables the analysis of gene expression profiles and the detection of fusion genes [109,110,111]. NGS-based technologies have important limitations for the detection of CNVs. For WGS, uneven coverage in different regions of the genome makes it difficult to obtain accurate breakpoints, and the lack of a reference normal sample makes data normalization difficult, which is particularly challenging in aneuploid genomes. With respect to RNA-seq, the relationship between copy number and gene expression levels is influenced by epigenetic regulation. Therefore, the detection of CNVs is mainly performed by chromosomal microarray analysis (CMA), which can include comparative genomic hybridization microarray or SNP microarray, which also allow the detection of CN-LOHs [109,110,111].

It is important to consider that no single method can detect different types of genetic variation with equal efficacy. Moreover, the broad coverage of WGS, WES, and RNA-seq increases the cost and the need for bioinformatics resources, limiting their clinical application. In contrast, targeted NGS panels represent a relatively inexpensive alternative that allows targeted analysis of clinically relevant regions of the genome with optimal depth and coverage, and the data obtained is manageable with fewer bioinformatics resources. Unfortunately, most of the currently commercially available panels are not fully applicable to the study of B-ALL, as they are designed for the study of solid tumors or adult hematological malignancies. Therefore, some research groups have opted to use engineered NGS panels or the combination of different genomic methods to maximize the detection of the different types of genetic variations associated with the disease [112,113,114,115].

## 6. Clinical Significance and Etiology of Variants

There are several bioinformatic algorithms for variant detection from genomic data, with varying degrees of sensitivity. A consensus on the application of these algorithms is yet to be achieved. A comprehensive data curation process is necessary to exclude numerous variants that represent false positives, common variants, and variants of unknown clinical significance. This process involves the use of prior knowledge of variants associated with B-ALL to identify clinically relevant alterations [10,11,21]. This process should be performed by qualified personnel and should follow the recommendations of the guidelines for variant interpretation in cancer. Automated search engines are available to consult the information available in various public databases, that include the population frequency of variants in groups of individuals with specific characteristics such as ethnicity, age, cancer type (e.g., GenomAD Genomes/Exomes, ClinVar, COSMIC), and the available evidence on the diagnostic, prognostic, or therapeutic value associated with the variants (OncoKB) [10,11,21]. This information facilitates the identification of pathogenic variants that are enriched in patients with the disease, exhibit high penetrance, and are of clinical significance in B-ALL or cancer in general. In contrast, benign or probably benign variants, which are prevalent in the general population, have low penetrance and are typically not associated with the disease. A prevalent finding is variants of uncertain significance (VUS), which do not satisfy the requisite criteria to be classified as benign variants. However, there is also an absence of substantial evidence to support their association with cancer [10,11,21].

VUS represent a significant challenge in clinical practice due to their inherent uncertainty, which can complicate decision-making processes and result in the application of inappropriate clinical treatments. Therefore, it is necessary to apply analytical resources focused on generating the necessary evidence to determine the biological and, ultimately, clinical role of these variants. The reclassification of these variants as benign or pathogenic is dependent upon this evidence. To differentiate between the potentially deleterious or neutral effects of VUS, an integrative analysis must be performed. This analysis should incorporate various characteristics of the variant, including its population frequency, the type of variant, its location in the functional product, its effect on function or stability, its degree of conservation across species, its presence in morphologically abnormal tissues, and its segregation among relatives. Unfortunately, all of this information is not always available, and no single type of evidence is sufficient to confidently predict or rule out the pathogenicity of a VUS [116,117].

Leukemic cells contain somatic and germline variants, and distinguishing the etiology of the variants can be a complex process. Somatic variants have been observed to manifest as leukemia-specific or to arise from clonal hematopoiesis (somatic mosaicism restricted to hematopoietic lineages). In contrast, germline variants are present in all cells of the organism and are heritable [118]. A variant should be suspected to be germline if it affects a proportion of cells greater than the leukemic blast population as determined by cytomorphology and/or flow cytometry. The proportion of the variant in the tumor can be inferred by examining the value of the allelic fraction (VAF) (in the case of NGS) or the Log2R and BAF values (in the case of CMA) [118]. Ideally, germline variants will have a VAF ∼0.5 if heterozygous, ∼1.0 if homozygous, or show concordant patterns between Log2R/BAF plots if present in all cells analyzed. However, when the biological sample is a pure tumor specimen, it becomes difficult to distinguish between the germline and somatic etiology of the variant, and the VAF may be altered by loss of heterozygosity, the presence of CNVs, and structural rearrangements, thus, altering its association with the zygosity of the variant. Therefore, a VAF > 0.3 may indicate the presence of a germline variant, whereas a VAF < 0.3 may indicate a somatic variant present in tumor subclones, a product of clonal hematopoiesis, or an artifact of NGS [118].

Suspicion of germline etiology is increased when the variant involves a cancer predisposition gene in a patient with clinical criteria for a hereditary cancer syndrome; in such cases, it is necessary to confirm the etiology of the variant in germline DNA [118]. In healthy individuals, germline DNA is commonly obtained from venous blood, buccal swabs, or hair bulbs; however, in B-ALL patients, such samples may be contaminated with tumor cells or have low yields due to common oncologic treatments. Best practices include the use of skin fibroblasts, although it should be noted that culturing these cells takes several weeks and can be technically demanding. The use of buccal mucosa-derived epithelial cells is also a reliable source of germline DNA when procedures to minimize leukocyte contamination are required [118,119].

## 7. Genetic Variants Applied to Clinical Practice

This section reviews three case reports of pediatric patients with B-ALL carrying somatic and germline pathogenic variants, in which variant characterization influenced disease management, provided an explanation for the observed phenotype, and/or prevented disease progression.

Case #1: Vatsayan et al., (2023) [120] reported a 12-year-old patient diagnosed with *CRLF2::P2RY8* B-ALL in 93% of cells, who was treated with the high-risk chemotherapy regimen AALL1131 (vincristine- and daunorubicin-based) [121]. Seven months later, he developed thyroid cancer. The presence of multiple neoplasms in less than one year suggested the possibility of a cancer predisposition syndrome. Family history revealed thyroid cancer in a maternal uncle. An NGS panel study of the patient revealed the pathogenic variant *CHEK2* p.T367Mfs*15. Fifteen months after diagnosis of B-ALL (maintenance), the patient relapsed in the central nervous system, and the bone marrow aspirate showed 15% blasts positive for *CRLF2::P2RY8*. The patient received intrathecal chemotherapy with vincristine and dexamethasone for several weeks until remission was achieved and was then lymphodepleted in preparation for CAR-T therapy. Bone marrow evaluation 30 days later was negative for MRD, and the patient remained in remission at subsequent evaluations every 3 to 6 months.

Comment: The review of the patient’s family history from the time of diagnosis of B-ALL could have anticipated the detection of the pathogenic variant in *CHEK2*. With this information, it may have been possible to adapt the therapeutic regimen or add Ruxolitinib, which has been used as a treatment for B-ALL positive for patients with *CRLF2::P2RY8* rearrangement [22,28]. The administration of a DNA-binding drug-based chemotherapy regimen likely contributed to the development of the patient’s second cancer and relapse of B-ALL. The efficacy of CAR-T can be attributed to its immunotherapeutic mechanism, which functions independently of the patient’s genetic characteristics, thereby ensuring broad applicability and significant therapeutic potential.

Case #2: Duployez et al., (2021) [74] reported a 17-year-old patient (proband) with an 11-year-old sister (II.1) and a 25-year-old brother (II.3), children of asymptomatic parents. All three siblings were diagnosed with B-ALL, negative for sentinel chromosomal abnormalities and positive for *CDKN2A* deletions and activating variants in RAS genes. After receiving chemotherapy, siblings II.1 and II.3 remained in remission, but the proband relapsed and was treated with allogeneic HSCT from sibling II.3 (in remission). The proband experienced a second relapse 20 years after HSCT and subsequently died. The sister II.1 remained in remission until 30 years after diagnosis and never received HSCT, and the brother II.3 relapsed and died 14 years post donation. WES was performed on bone marrow DNA from different disease time points of all 3 siblings, as well as on peripheral blood from both parents. The likely pathogenic variant PAX5p.R38H, with VAF 50%, was detected in all the bone marrow samples from the patients and in the peripheral blood of one parent [74].

Comment: The following three factors provide substantial evidence for the hypothesis that hereditary B-ALL is associated with *PAX5* deficiency: (1) the presence of a likely pathogenic variant in *PAX5* (a B-ALL high-susceptibility gene), (2) a 50% VAF observed in samples from most of the family members, strongly suggesting a germline etiology, and (3) the diagnosis of B-ALL in several family members. According to the literature, treatment with intensive chemotherapy improves EFS in patients with *PAX5*-altered B-ALL, as evidenced in the sister II.1. For the proband, it is not specified whether the donor’s germline status was determined prior to HSCT; however, it is essential to know this information to avoid failure of HSCT therapy.

Case #3: Lin et al. (2019) [122] reported a patient with B-ALL positive for somatic deletions in the *IKZF1* and *CDKN2A* genes detected by CMA. This patient also inherited two germline variants from both healthy parents: the probably pathogenic JAK2 p.Gly571Ser variant, from heterozygous carrier father, and VUS STAT3 p.Lys370Arg, from heterozygous carrier mother. There was no family history of cancer, and both variants have a population frequency of less than 0.01. JAK2 and STAT3 encode proteins of the JAK-STAT pathway. Both variants are located in the catalytic domains of the corresponding proteins. Functional assays using in vitro lymphoid models revealed that the variants do not possess oncogenic properties when present in isolation, but they promote independent proliferation stimuli when coexisting.

Comment: The patient’s somatic deletions are compatible with the *IKZF1^plus^* profile, which confers an unfavorable prognosis. It is recommended to consider intensive chemotherapy and monitoring for minimal residual disease. The addition of Ruxolitinib to chemotherapy could be effective against hyperactivation of the JAK-STAT pathway caused by the presence of germline variants in genes of this pathway. The study of the properties of VUS revealed that this variant acts synergistically with the pathogenic variant in *JAK2* to promote leukemogenesis. This case exemplifies the importance of analyzing VUS to determine its biological and clinical relevance.

## 8. Future Directions

The implementation of pathogenic variants in the clinical management of B-ALL requires the collaborative efforts of medical oncologists, geneticists, and specialists in the interpretation of genomic data. Despite the noteworthy advancements in the field of somatic and germline characterization, further exploration into genetic variation remains imperative. This encompasses the use of different genomic approaches, which allow a complete characterization of the diverse molecular classes of pathogenic variants. However, the application of these approaches is limited by their relatively high cost and the difficulty of interpreting bioinformatic data. Additionally, comprehensive characterization of VUS is necessary to improve our understanding of the pathological processes associated with B-ALL. This requires a high-cost effort for sustained reclassification. Addressing these research gaps can facilitate the implementation of precision medicine for patients with this disease.

## Data Availability

Not applicable.

## Supplementary Materials

The following supporting information can be downloaded at: https://www.mdpi.com/article/10.3390/biomedicines13071626/s1, Table S1: Common kinase gene rearrangements associated with Ph-like B-ALL. Table S2: Common gene fusions involving transcription factor genes associated with specific B-ALL molecular subtypes. Figure S1: (**A**). Schematic representation of the functional effect of CRLF2 rearrangements. (**B**). Schematic representation of JAK/ABL class gene fusions. (**C**). Principal Ph-like activating point mutations in CRLF2, JAK2, NRAS and KRAS genes. Supplementary [123]. Figure S2: (**A**). The UBTF::ATXNTL3 and CDX2 rearrangements. (**B**). Somatic point mutations in transcription factor genes associated with B-ALL molecular subtypes. (**C**). The DUX4/ERG rearrangements. Figure S3. Somatic pathogenic variants commonly associated with chemoresistance in B-ALL. Figure S4. Characteristic germline pathogenic variants associated with B-ALL susceptibility.

## Abbreviations

The following abbreviations are used in this manuscript:

ACMG	American college of genetics and genomic medicine
AD	Autosomal dominant inheritance
ALL	Acute lymphoblastic leukemia
AMP	Association of molecular pathology
ASCO	American society of clinical oncology
BAF	B allele frequency
B-ALL	B-cell precursor acute lymphoblastic leukemia
CAR-T	Chimeric antigen receptor therapy
CAP	College of American pathologist
CGC	Cancer genomic consortium
CNV	Copy number variant
CN-LOH	Copy neutral loss of heterozygosity
CMA	Chromosomal microarray analysis
CMMRD	Constitutional mismatch repair deficiency syndrome
COG	Children oncology group
EFS	Event-free survival
FDA	Food and drug administration
FISH	Fluorescence in situ hybridization
HR	High-risk
HSCT	Hematopoietic stem cell transplant
LFS	Li Fraumeni syndrome
MRD	Minimal residual disease
NCI	National cancer institute
NFS	Neurofibromatosis syndrome
NLS	Noonan/Leopard syndrome
NGS	Next-generation sequencing
NJS	Nijmegen syndrome
RT-PCR	Reverse transcript polymerase chain reaction
SNV	Single nucleotide variant
SR	Standard risk
TKI	Tyrosine kinase inhibitor
VAF	Variant allele fraction
VUS	Variant of uncertain significance
WES	Whole exome sequencing
WBC	White blood cell
WHO	World health organization
WGS	Whole genome sequencing
