# Supplementary material for: A Comprehensive Review of Somatic and Germline Biomarkers Associated with Childhood B-Cell Precursor Acute Lymphoblastic Leukemia: From Biological Significance to Precision Medicine Opportunities
Source: Biomedicines. 2025 Jul 2;13(7):1626. doi: 10.3390/biomedicines13071626 (PMC12293005; doi:10.3390/biomedicines13071626)
Supplement: Supplementary file 1 [file biomedicines-13-01626-s001.zip › biomedicines-3668022-supplementary.pdf]

SUPPLEMENTARY MATERIAL

**Supplementary Table S1**<br>Common kinase gene rearrangements associated with Ph-like B-ALL		
	***CRLF2* abnormalities** [1–4]	
IGH::CRLF2<br>translocation	*P2RY8::CRLF2*<br>fusion by microdeletion	c.695T>G p.Phe232Cys<br>point mutation
	**JAK-STAT class gene fusions** [1–4]	
	JAK2 3´ partners	
ATF7IP	*PAX5*	*TERF2*
BCR	*PCM1*	*TRP*
EBF1	*PPFIBP1*	*USP25*
ETV6	*RFX3*	*ZBTB46*
GOLGA5	*SMU1*	*ZNF27A*
HMBOX1	*SNX29*	*ZNF340*
OFD1	*SSBP2*	
	STRN3	
	EPOR partners	
	LAIR1, IGH, IGK, IGL, THADA	
	TYK2 5´ partners	
	MYB, SMARCA4, ZNF340	
	**ABL class gene fusions** [1–4]	
	ABL1 5´ partners	
ATF7IP	*PAX5*	*STRN3*
BCR1	*PCM1*	*TERF2*
EBF1	*PPFIBP1*	*TRP*
ETV6	*RFX3*	*USP25*
GOLGA5	*SMU1*	*ZBTB46*
HMBOX1	*SNX29*	*ZNF27A*
OFD1	*SSBP2*	*ZNF340*
	ABL2 5´ partners	
	PAG1, RCSD1, ZC3HAV1	
	CSF1R 5´ partners	
	MEF2D, SSBP2, TBL1XR1	
	PDGFRB 5´ partners	
	ATF7IP, EBF1, ETV6, NUMA1, SNX29, SSBP2, TERF2, TNIP1, ZEB2, ZMYND8, ZNF608	

Other kinase gene fusions [1–4]
PTK2B *3´* partners
KDM6A, STAG2, TMEM2
NTRK3::ETV6
BCR::FGFR1
ZMYM2::FLT3
DNTT::BLNK
KANK1::CBL
ZFAND3::DGKH

Supplementary Figure S1A. Schematic representation of the functional effect of *CRLF2* rearrangements. The coding region of *CRLF2* is under the transcriptional control of *P2RY8* (by interstitial deletion) or *IGH* (by cryptic translocation). This leads to overexpression of mRNA and protein [1–4].

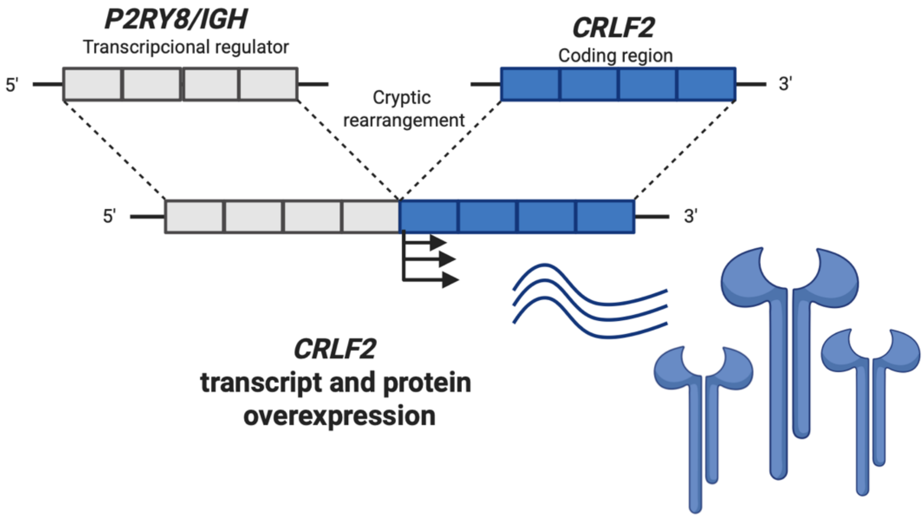

Supplementary Figure S1B. Schematic representation of JAK/ABL class gene fusions. Each gene fusion contains the 5' partner and the 3' region of the kinase gene with intact kinase domain (KD). This results in chimeric transcripts and proteins that deregulates JAK or ABL signaling [1–4].

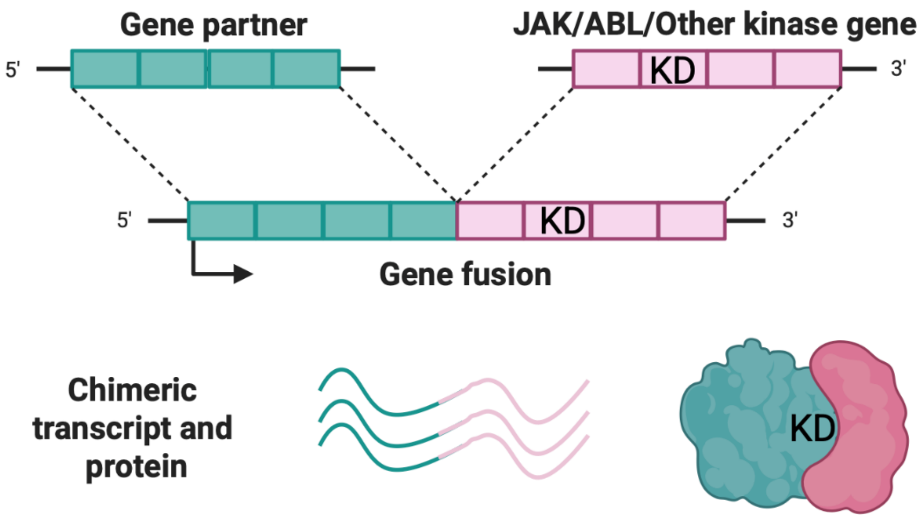

Supplementary Figure S1C. Principal Ph-like activating point mutations in *CRLF2, JAK2, NRAS* and *KRAS* genes [1–5].

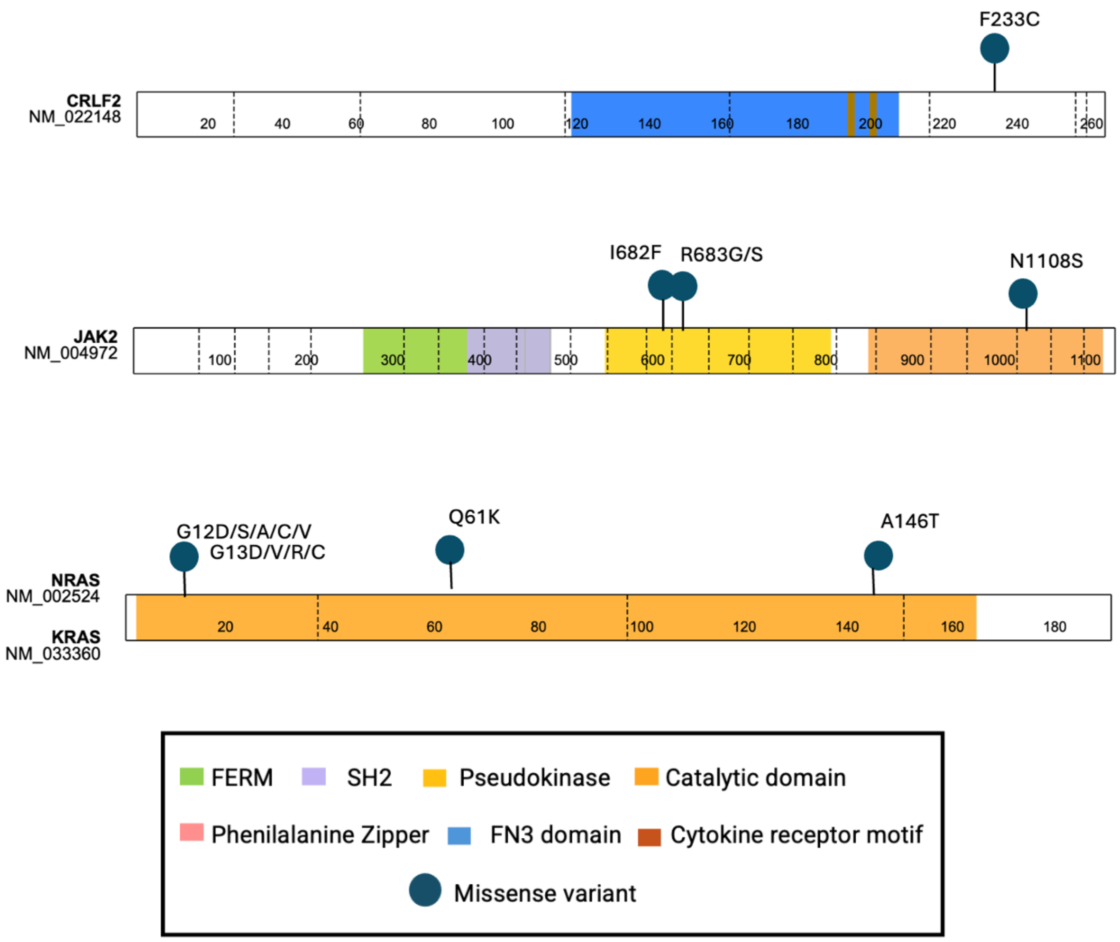

Supplementary Table S2. Common gene fusions involving transcription factor genes associated to specific B-ALL molecular subtypes

***PAX5* partners** [6]		
AUTS1	*FOXP1*	*SEC1P*
BCOR	*GREB1L*	*KANK1*
CBFA2T2	*MBNL1*	*MLLT3*
DACH1	*NCOA5*	*GRM3*
DACH2	*NOL4L*	*MPRIP*
DBX1	*RHOXF2*	*ZNF521*
ELN	*TAF3*	*LINC01400*
ETV6	*MAPT*	*ZCCHC7*
FBRSL1		
***ETV6* partners in *ETV6::RUNX1* like subtype** [7,8]		
PMEL, IKZF1, CDK2, CREBBP, BCL2L14, BORCS5, MSH6		
***ZNF384* partners** [8]		
ARID1B, BMP2K, CREBBP, EP300, EWSR1, SMARCA2, SYNGR, TAT15, TCF3		
MEF2D partners [9]		
BCL9, CSF1R, DAZAP1, FOXJ2, HNRNPUL1, HNRNPH1, SS18		
NUTM1 partners [10]		
MYB, SMARCA4, ZNF340		

Supplementary Figure S2A. The *UBTF::ATXNTL3* and *CDX2* rearrangements

1. Schematic representation of the *UBTF::ATXNTL3* rearrangement caused by interstitial deletion, resulting in a chimeric transcript. **2.** The *PAN3/FLT3* enhancer is a regulator of gene expression. **3.** A microdeletion relocates the *PAN3/FLT3* enhancer near *CDX2*, leading to its ectopic expression [11].

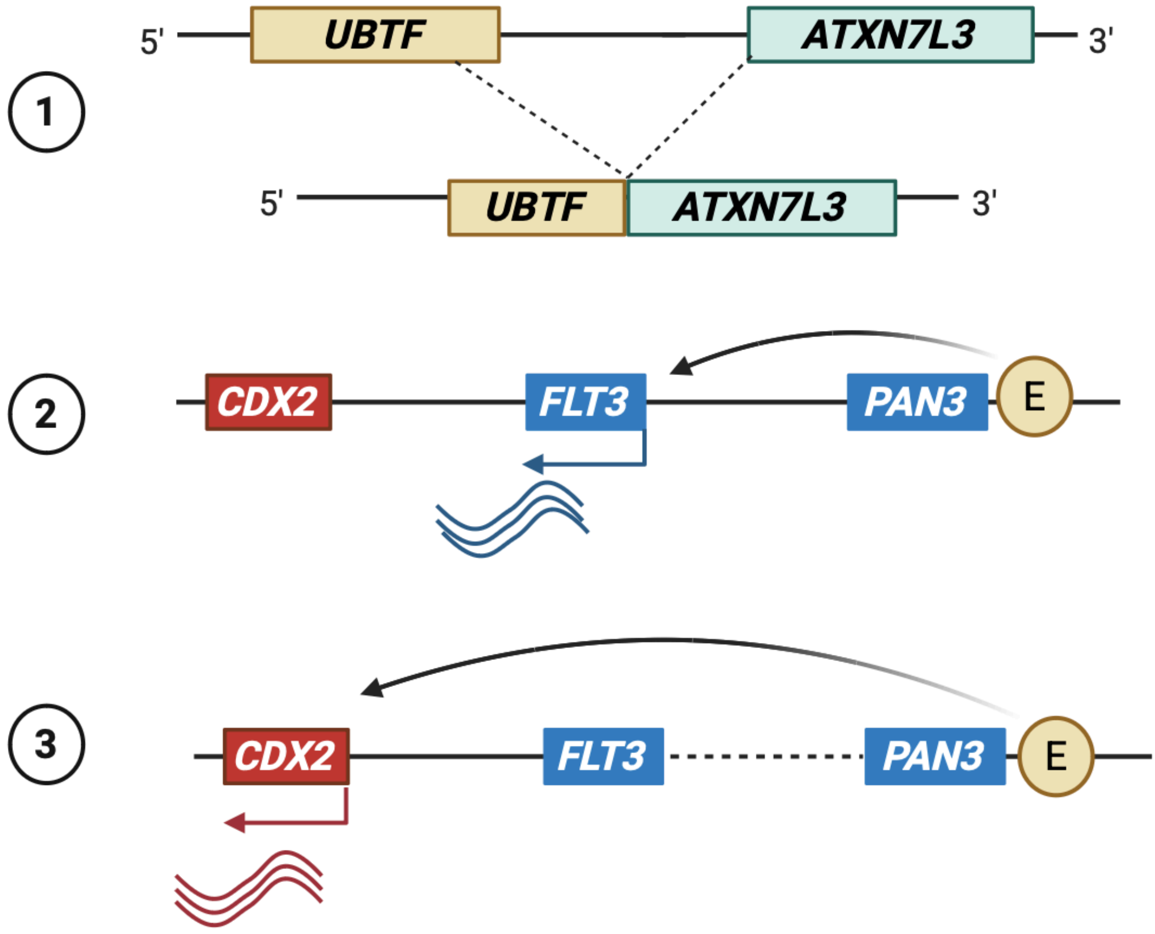

Supplementary Figure S2B. Somatic point mutations in transcription factor genes associated with B-ALL molecular subtypes [5,6,12–14]

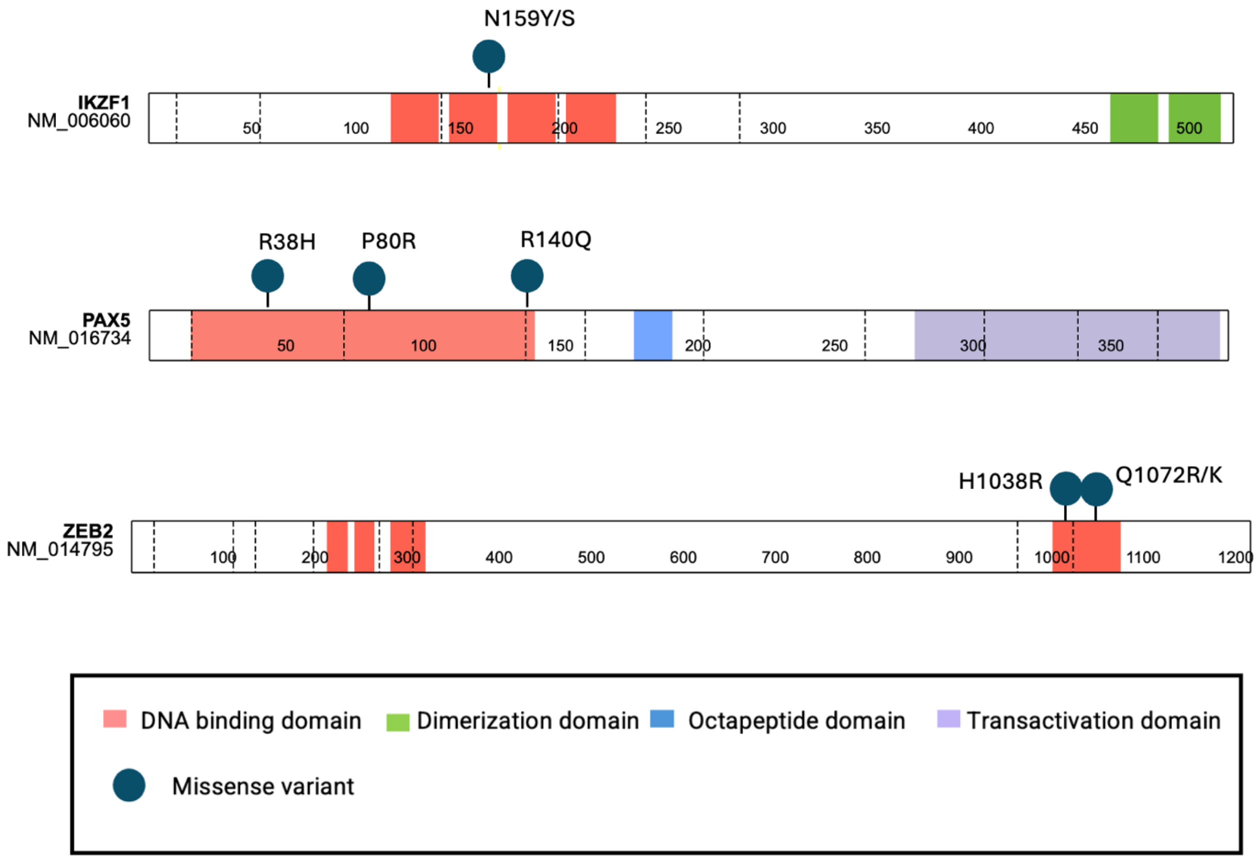

Supplementary Figure S2C. The *DUX4/ERG* rearrangements

1. The *DUX4* gene is located between the *D4Z4* repeats and is partially inserted into the *IGH* locus. **2**. The truncated DUX4 isoform is overexpressed. **3.** The truncated DUX4 isoform has aberrant activity, resulting in the transcription of a truncated ERG isoform. **4.** The truncated *ERG* isoform also has an aberrant activity that favours the deletion of the wild-type *ERG* allele [1,15]

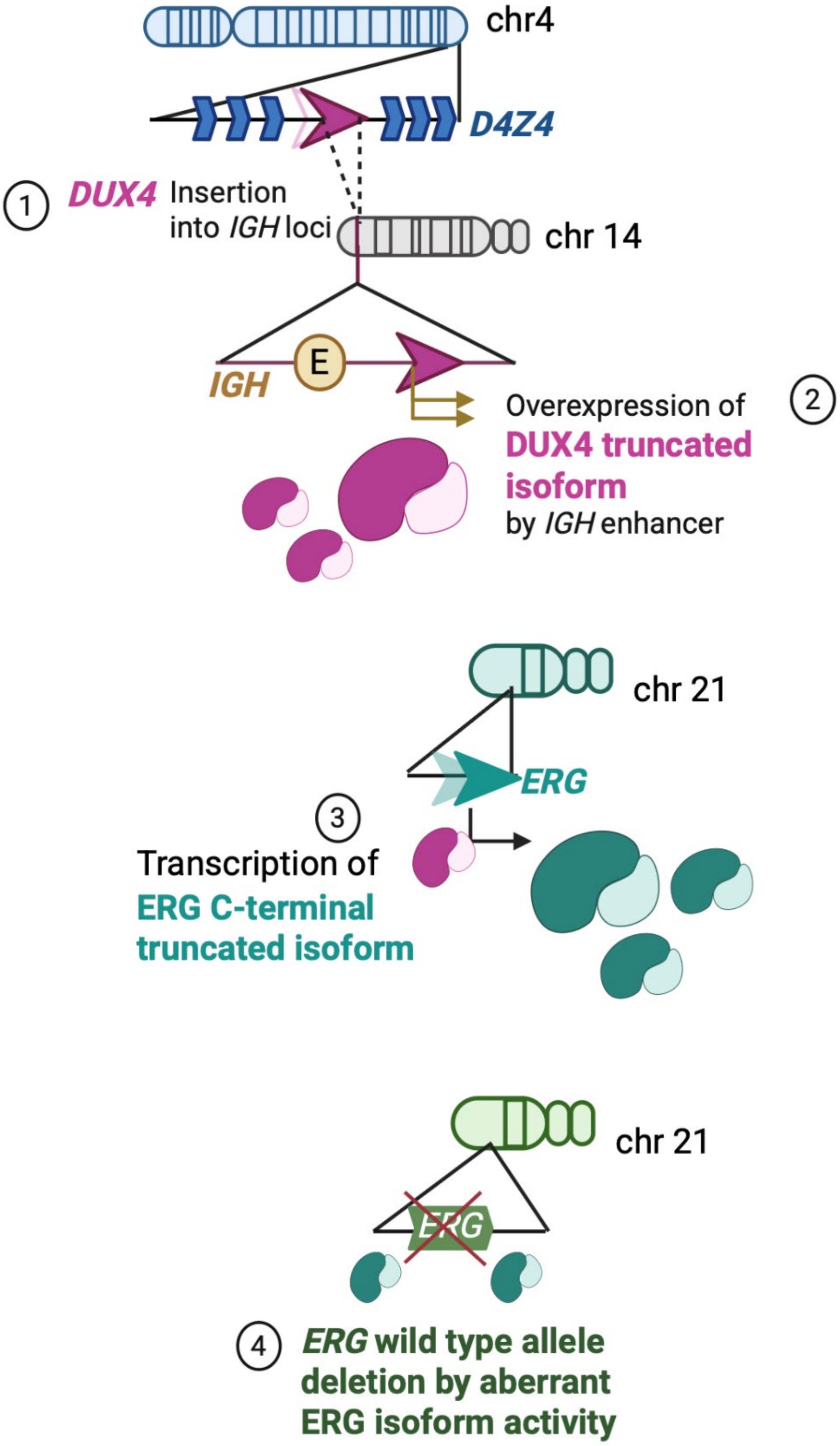

Supplementary Figure S3. Somatic pathogenic variants commonly associated with chemoresistance in B-ALL [5,16–18]

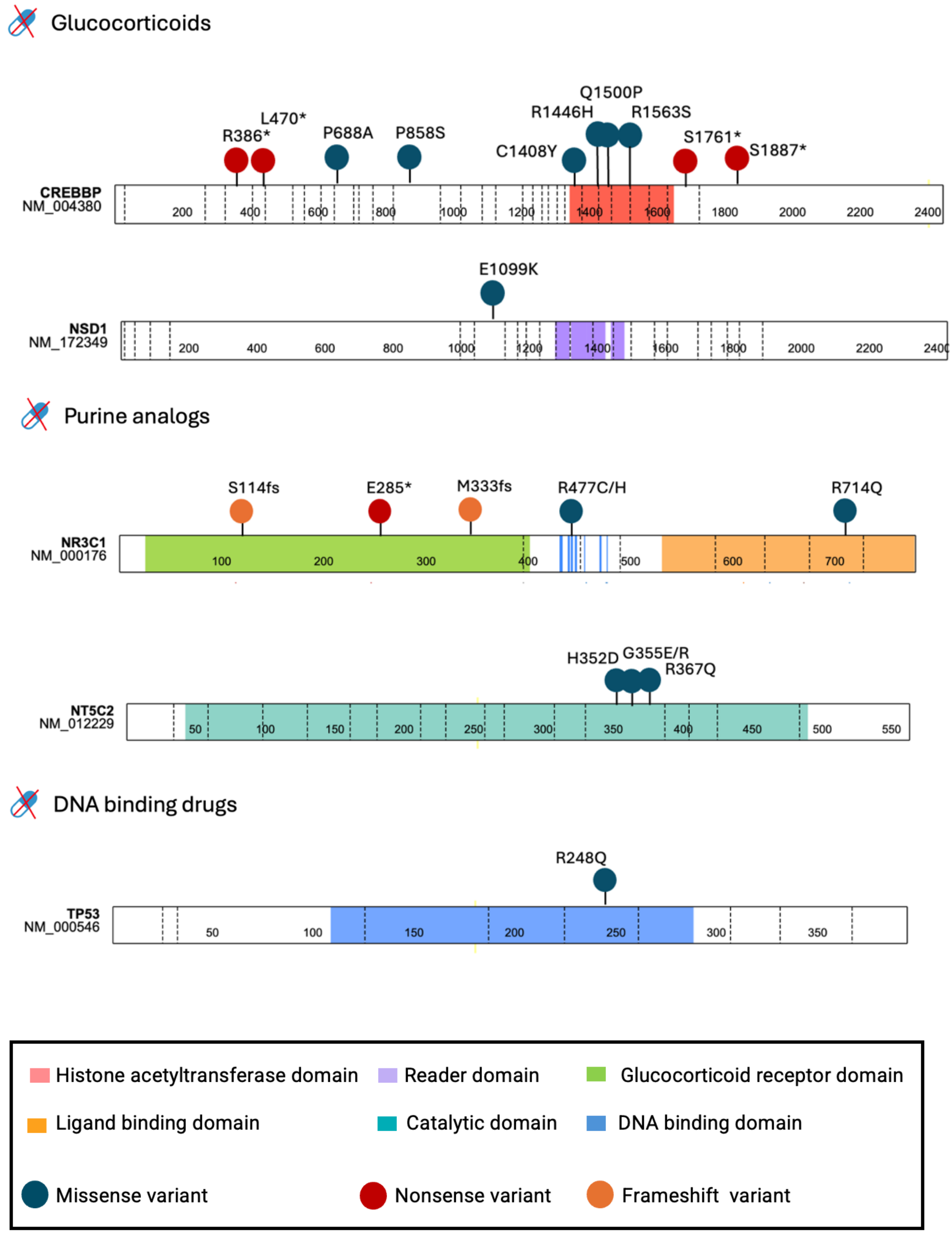

Supplementary Figure S4. Characteristic germline pathogenic variants associated with B-ALL susceptibility [5,19–25]

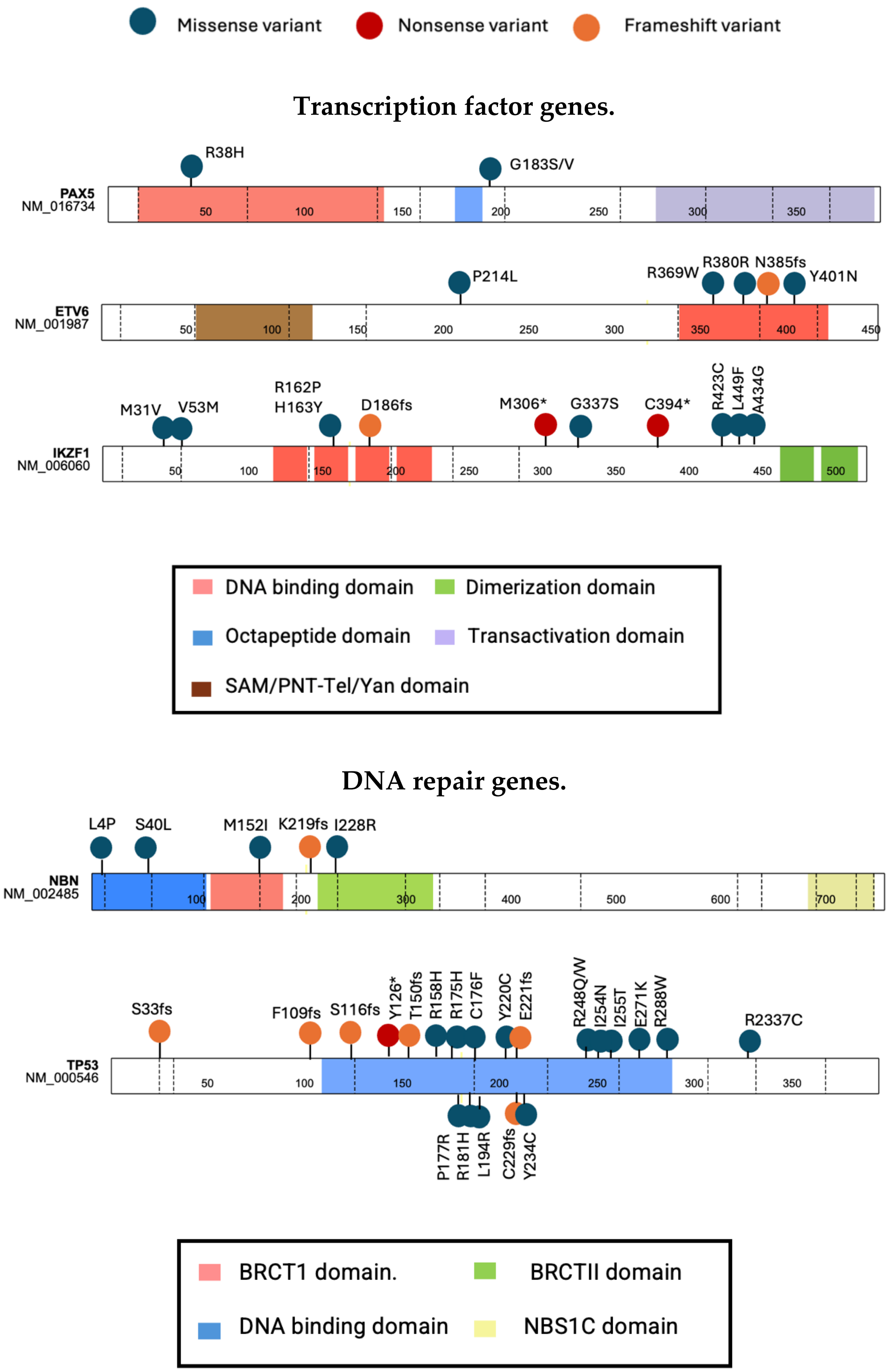

References.

1. Iacobucci I, Mullighan CG. Genetic Basis of Acute Lymphoblastic Leukemia. *J Clin Oncol Off J Am Soc Clin Oncol*. 2017;35(9):975-983. doi:10.1200/JCO.2016.70.7836

2. Roberts KG, Li Y, Payne-Turner D, et al. Targetable kinase-activating lesions in Ph-like acute lymphoblastic leukemia. *N Engl J Med*. 2014;371(11):1005-1015. doi:10.1056/NEJMoa1403088

3. Tran TH, Hunger SP. The genomic landscape of pediatric acute lymphoblastic leukemia and precision medicine opportunities. *Semin Cancer Biol*. 2022;84:144-152. doi:10.1016/j.semcancer.2020.10.013

4. Yokota T, Kanakura Y. Genetic abnormalities associated with acute lymphoblastic leukemia. *Cancer Sci*. 2016;107(6):721-725. doi:10.1111/cas.12927

5. McLeod C, Gout AM, Zhou X, et al. St. Jude Cloud: A Pediatric Cancer Genomic Data-Sharing Ecosystem. *Cancer Discov*. 2021;11(5):1082-1099. doi:10.1158/2159-8290.CD-20-1230

6. Gu Z, Churchman ML, Roberts KG, et al. PAX5-driven subtypes of B-progenitor acute lymphoblastic leukemia. *Nat Genet*. 2019;51(2):296-307. doi:10.1038/s41588-018-0315-5

7. Lilljebjörn H, Henningsson R, Hyrenius-Wittsten A, et al. Identification of ETV6-RUNX1-like and DUX4-rearranged subtypes in paediatric B-cell precursor acute lymphoblastic leukaemia. *Nat Commun*. 2016;7:11790. doi:10.1038/ncomms11790

8. Lilljebjörn H, Fioretos T. New oncogenic subtypes in pediatric B-cell precursor acute lymphoblastic leukemia. *Blood*. 2017;130(12):1395-1401. doi:10.1182/blood-2017-05-742643

9. Ohki K, Kiyokawa N, Saito Y, et al. Clinical and molecular characteristics of MEF2D fusion-positive B-cell precursor acute lymphoblastic leukemia in childhood, including a novel translocation resulting in MEF2D-HNRNPH1 gene fusion. *Haematologica*. 2019;104(1):128-137. doi:10.3324/haematol.2017.186320

10. Hormann FM, Hoogkamer AQ, Beverloo HB, et al. NUTM1 is a recurrent fusion gene partner in B-cell precursor acute lymphoblastic leukemia

associated with increased expression of genes on chromosome band 10p12.31-12.2. *Haematologica*. 2019;104(10):e455-e459. doi:10.3324/haematol.2018.206961

11. Passet M, Kim R, Gachet S, et al. Concurrent CDX2 cis-deregulation and UBTF::ATXN7L3 fusion define a novel high-risk subtype of B-cell ALL. *Blood*. 2022;139(24):3505-3518. doi:10.1182/blood.2021014723

12. Duffield AS, Mullighan CG, Borowitz MJ. International Consensus Classification of acute lymphoblastic leukemia/lymphoma. *Virchows Arch Int J Pathol*. 2023;482(1):11-26. doi:10.1007/s00428-022-03448-8

13. Zaliova M, Potuckova E, Lukes J, et al. Frequency and prognostic impact of ZEB2 H1038 and Q1072 mutations in childhood B-other acute lymphoblastic leukemia. *Haematologica*. 2021;106(3):886-890. doi:10.3324/haematol.2020.249094

14. Li JF, Dai YT, Lilljebjörn H, et al. Transcriptional landscape of B cell precursor acute lymphoblastic leukemia based on an international study of 1,223 cases. *Proc Natl Acad Sci U S A*. 2018;115(50):E11711-E11720. doi:10.1073/pnas.1814397115

15. Rehn JA, O'Connor MJ, White DL, Yeung DT. DUX Hunting-Clinical Features and Diagnostic Challenges Associated with DUX4-Rearranged Leukaemia. *Cancers*. 2020;12(10):2815. doi:10.3390/cancers12102815

16. Mullighan CG, Zhang J, Kasper LH, et al. CREBBP mutations in relapsed acute lymphoblastic leukaemia. *Nature*. 2011;471(7337):235-239. doi:10.1038/nature09727

17. Li B, Brady SW, Ma X, et al. Therapy-induced mutations drive the genomic landscape of relapsed acute lymphoblastic leukemia. *Blood*. 2020;135(1):41-55. doi:10.1182/blood.2019002220

18. Pierro J, Saliba J, Narang S, et al. The NSD2 p.E1099K Mutation Is Enriched at Relapse and Confers Drug Resistance in a Cell Context-Dependent Manner in Pediatric Acute Lymphoblastic Leukemia. *Mol Cancer Res MCR*. 2020;18(8):1153-1165. doi:10.1158/1541-7786.MCR-20-0092

19. Duployez N, Jamrog LA, Fregona V, et al. Germline PAX5 mutation predisposes to familial B-cell precursor acute lymphoblastic leukemia. *Blood*. 2021;137(10):1424-1428. doi:10.1182/blood.2020005756

20. Hyde RK, Liu PP. Germline PAX5 mutations and B cell leukemia. *Nat Genet*. 2013;45(10):1104-1105. doi:10.1038/ng.2778

21. Feurstein S, Godley LA. Germline ETV6 mutations and predisposition to hematological malignancies. *Int J Hematol*. 2017;106(2):189-195. doi:10.1007/s12185-017-2259-4

22. Di Paola J, Porter CC. ETV6-related thrombocytopenia and leukemia predisposition. *Blood*. 2019;134(8):663-667. doi:10.1182/blood.2019852418

23. Churchman ML, Qian M, Te Kronnie G, et al. Germline Genetic IKZF1 Variation and Predisposition to Childhood Acute Lymphoblastic Leukemia. *Cancer Cell*. 2018;33(5):937-948.e8. doi:10.1016/j.ccell.2018.03.021

24. Escherich C, Chen W, Li Y, et al. Germline Genetic NBN Variation and Predisposition to B-cell Acute Lymphoblastic Leukemia in Children. *Res Sq*. Published online July 21, 2023:rs.3.rs-3171814. doi:10.21203/rs.3.rs-3171814/v1

25. Qian M, Cao X, Devidas M, et al. TP53 Germline Variations Influence the Predisposition and Prognosis of B-Cell Acute Lymphoblastic Leukemia in Children. *J Clin Oncol Off J Am Soc Clin Oncol*. 2018;36(6):591-599. doi:10.1200/JCO.2017.75.5215
